# Hypoxia induces ferroptotic cell death mediated by activation of the inner mitochondrial membrane fission protein MTP18/Drp1 in invertebrates

**DOI:** 10.1016/j.jbc.2025.108326

**Published:** 2025-02-18

**Authors:** Jiaqi Liu, Xichao Sun, Yijie Wu, Zhimin Lv, Na Zhou, Chao Bian, Shengming Sun

**Affiliations:** 1 Key Laboratory of Exploration and Utilization of Aquatic Genetic Resources, Ministry of Education, Shanghai Ocean University, Shanghai, China; 2 International Research Center for Marine Biosciences at Shanghai Ocean University, Ministry of Science and Technology, Shanghai, China; 3 School of Pharmacy and State Key Laboratory for Quality Research of Chinese Medicines, (R & D Center) Lab. for Drug Discovery from Natural Resource,Macau University of Science and Technology, Taipa, Macau, China; 4 College of Life and Marine Sciences, Shenzhen University, Shenzhen, China

**Keywords:** Drp1, ferroptosis, hypoxia, invertebrate, mitochondrial fission

## Abstract

Hypoxia and ischemia damage sensitive organelles such as mitochondria, and mitochondrial dysfunction contributes to metabolic disorders in crustaceans under hypoxia. The mechanisms associated with ferroptosis in hypoxic disorders have not been determined in crustaceans. In particular, the early molecular events of mitochondrial dynamics in crustaceans require clarification. In this study, two evolutionarily conserved mitochondrial fission proteins, Drp1 and MTP18, were identified in oriental river prawn (*Macrobrachium nipponense*). *In vitro*, ferroptosis-mediated impairment of mitochondrial membrane potential was induced by hypoxia in oriental river prawn hemocytes. In hypoxia-induced hemocytes, activation of Drp1 by increased phosphorylation at S616 was identified. Drp1 mitochondrial translocation also increased, and mitochondrial fusion-related protein expression decreased *in vivo*. Altered mitochondrial fission-fusion dynamics have been linked to mitochondrial dysfunction, inducing a classic ferroptosis mechanism. *Marf* overexpression or *Drp1* knockdown protected against mitochondrial dysfunction and ferroptotic cell death *in vitro*. Furthermore, hypoxia-induced mitochondrial fission was verified to be driven by Drp1/MTP18 interaction. Under hypoxia, *MTP18* transcription was increased by the binding of activated HIF-1α to hypoxia response elements in its promoter. Conjointly, *MTP18* knockdown resulted in less apoptosis and decreased prawn mortality in gill tissue *in vitro*, suggesting that adaptation to hypoxia involves a vital function by *MTP18*. In conclusion, we uncovered a conserved role of mitochondrial fission in hypoxia-induced ferroptotic cell death. Therefore, we suggest that specific modulation of MTP18/DRP1-mediated mitochondrial dynamics might be a potential therapeutic strategy in hypoxic stress-induced tissue injury in invertebrates.

Oxygen is far less abundant in water than in air, and its diffusion rate in water is estimated to be 1/10,000 of that in air (1). Environmental hypoxia (low oxygen levels) in aquatic ecosystems is a widespread problem. In large lakes and oceans, hypoxic areas (“dead zones”) have increased dramatically, in which the dissolved oxygen (DO) concentration is frequently less than 2 ml of O_2_/l (2, 3). In fact, hypoxia presents a significant risk for the growth and survival of aquatic organisms because most aquatic organisms are sensitive to hypoxia (4). Among species, the hypoxia threshold varies, *e.g.*, most crustaceans have a higher half-maximal lethal concentration (LC_50_) of DO than fish. Hypoxia-inducible factor 2 alpha (HIF-2α) plays a role in the altered mitochondrial dynamics and remodeling in mammals under hypoxia; however, so far, an HIF-2α gene ortholog has not been found in crustacean genomes (4). Although several studies have investigated the chronic and acute effects of hypoxia on the physiological response of aquatic crustaceans, their mechanism of hypoxia adaptation involving the hypoxia-inducible factor 1 (HIF-1) pathway has received little research attention compared with that in mammals (5). Hypoxic conditions induced binding of the transcription factor HIF-1 to hypoxia response elements (HRE) in gene promoters, thereby activating the expression of many genes involved in various cell activities, including metabolic reprogramming and cell apoptosis or survival (6, 7).

Ferroptosis is one of the oldest forms of regulated cell death from plants to animals (8). It differs from other classical types of nonapoptotic cell death by its biochemical characteristics of iron, lethal lipid reactive oxygen species (ROS) accumulation, and mitochondrial-morphology shrinkage with condensed mitochondrial membrane densities (9). Indeed, according to previous publications, hypoxia-induced mitochondrial dysfunction and ferroptosis are regulated by multiple pathways. Firstly, hypoxia can regulate ferroptosis in specific cells and conditions through a HIF-1-dependent mechanism (10). In particular, HIF-1α antagonizes ferroptosis by upregulating lipid metabolism-related genes (*e.g.*, those encoding fatty acid binding protein 3 and fatty acid binding protein 7) in cancer cells (11). Meanwhile, HIF-1 increases the transcription of solute carrier family 7 member 11 (SLC7A11), which protects cells from ferroptosis by decreasing ROS and increasing GSH (12, 13). Secondly, it is believed that mitochondrial fission and fusion are intimately related to ferroptosis (14). The nuclear translocation of HIF-1 is responsible, directly or indirectly, for mitochondrial dynamic regulation (15, 16, 17). Mitochondrial fission, fusion, and cristae remodeling allow the cell to fine-tune its energy production, suggesting that adaptation to hypoxia involves adjustment of cellular mitochondrial function and regulation of ferroptosis (17). The vast majority of invertebrates have an open circulatory system, and their body fluids (referred to as hemolymph, not blood) also leave the heart through arteries, but do not flow into capillaries. Hemocytes need to consume large amounts of ATP to defend against pathogens; therefore, they have a large mitochondrial distribution. However, it is unclear whether mitochondrial dysfunction and ferroptosis are involved in hypoxic stress-induced damage to crustacean hemocytes.

In cells, mitochondria are the main oxygen consumers. As a result, lower availability of oxygen severely affects mitochondrial functions (18). Under hypoxia, mitochondrial production of ATP is reduced; thus, glycolysis might still be considered as the main compensatory energy-producing system. It is likely that the mechanisms by which mitochondria are adapted to hypoxia evolved to maintain the energy supply. As highly dynamic organelles, mitochondria permanently undergo rounds of fusion and fission; however, under hypoxic conditions, mitochondria undergo fission and mitophagy to eliminate damaged mitochondria and then produce excess ROS *via* a decrease in respiratory activity (19). Although the cellular response to hypoxia needs to be rapid in order to survive, it seems likely that mitochondrial dysfunction is required “upstream” to promote cell apoptosis (20).

Mitochondria are dynamic organelles that constantly change their shape through fission and fusion. The fission-fusion cycle is dynamically balanced under normal conditions (21). In mammals, fusion of mitochondria is mediated by several dynamin-related GTPases such as mitofusin 1 (MFN1), mitofusin 2 (MFN2), and optic atrophy protein 1. In *Drosophila*, mitochondrial assembly regulatory factor (Marf) is an ortholog of mammalian MFN2 (22). Crustaceans and insects both belong to the phylum Arthropoda; thus, we also found that *Marf* exists in crustacean genomes (23). In the case of mitochondrial fission, dynamin-related protein 1 (Drp1) and mitochondrial fission process protein 1 (MTP18, also known as MTFP1) are evolutionarily conserved proteins found in simple to complicated animals, which have an important regulatory effect on mitochondrial fission. Among them, Drp1 mediates mitochondrial fission by assembling onto the surface of mitochondria and constricting this tubular organelle (19). Phosphorylation of DRP1 at serine 616 (pDRP1S616) promotes its translocation and binding to the outer mitochondrial membrane and mediates mitochondrial fragmentation (24). The abnormal activation of Drp1 induces excessive mitochondrial fragmentation, leading to mitochondrial damage (25, 26). However, it is unclear whether ferroptosis in response to hypoxia involves a failure of mitochondrial quality control, particularly the fission process.

Mitochondrial dynamics has emerged as a critical mechanism for cellular function and differentiation under hypoxic conditions. However, the role of mitochondrial dynamics in hypoxia-induced ferroptosis remains to be determined. The present study hypothesized that hypoxia activates higher expression of Drp1/MTP18, and phosphorylated Drp1 can promote a mitochondrial dynamic imbalance together with ferroptosis. To verify this hypothesis, *Drp1* and *MTP18* expression were knocked down using siRNAs in prawn hemocytes. Our findings demonstrated that HIF-1α-induced MTP18 promotes Drp1's role in excess mitochondrial fission under hypoxia. In addition, overexpression of *Marf* attenuated excess mitochondrial fission and reduced mitochondrial dysfunction. Mechanistically, our findings reveal a causal link among MTP18/Drp1 expression, increased mitochondrial fragmentation, mitochondrial dysfunction, and ferroptosis. Our work will serve as a foundation for further investigations into the roles of MTP18/Drp1 activation induced by hypoxia.

## Results

### Mitochondrial dysfunction and ferroptosis occur in prawn hemocytes under hypoxia exposure *in vitro*

**Figure 1:**
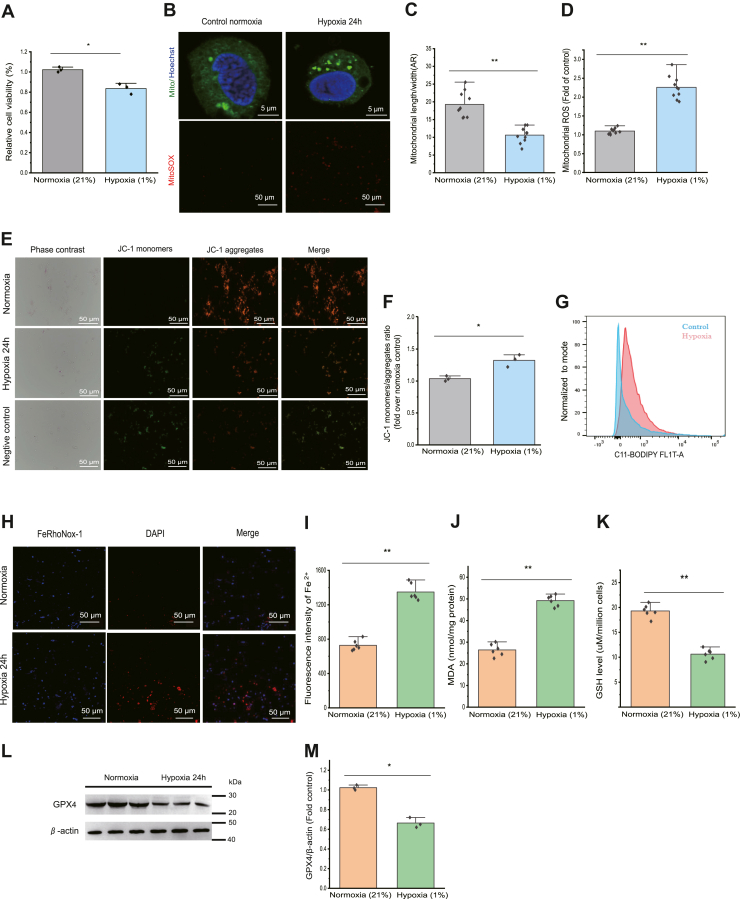
**Ferroptotic stressoccurs in primary cultured hemocytes from prawns upon hypoxic exposure *in vitro*.***A*, cell viability of primary cultured hemocytes treated with hypoxia. *B*–*D*, alterations to mitochondrial fission, the mitochondrial AR, and mitochondrial ROS production levels. The error bars indicate the mean ± SD (n = 10). *E*, representative images of mitochondrial membrane potential stained using JC-1, and a negative control was set up using CCCP as a proton gradient uncoupling agent. *F*, quantification of the mitochondrial membrane potential. The error bars indicate the mean ± SD (n = 3). *G*, lipid peroxidation was assessed in hemocytes using flow cytometry with the fluorescent probe C11-BODIPY. Plots displaying the mean percentages ± SD of positive cells relative to the total cell population are depicted. *H* and *I*, intracellular Fe^2+^ levels of hemocytes treated with hypoxia for 24 h was detected using a FeRhoNox-1 probe, and the fluorescence intensity of Fe^2+^ was quantified. The error bars indicate the mean ± SD (n = 6). *J*, MDA levels were measured to assess lipid peroxidation in hemocytes. *K*, GSH levels were determined using a GSH assay kit. The error bars indicate the mean ± SD (n = 6). *L* and *M*, Western blotting was performed to determine the level of GPX4 in hemocytes under hypoxia for 24 h. The error bars indicate the mean ± SD (n = 3). ∗*p* < 0.05 and ∗∗*p* < 0.01 (two-tailed unpaired Student’s *t* test) for control normoxia (21%) *versus* hypoxia (1%)-treated primary cultured hemocytes. AR, aspect ratio; CCCP, carbonyl cyanide-m-chlorophenylhydrazone; GPX4, glutathione peroxidase 4; MDA, malondialdehyde; ROS, reactive oxygen species.

Hemolymph, identified as an oxygen transportation system in crustaceans, plays a crucial role in response to hypoxic stress because there are many hemocytes to carry oxygen to organs and tissues. Herein, the results showed that cell viability in hemocytes was inhibited during hypoxia (Fig. 1*A*). Mitochondrial morphological changes in hemocytes in response to hypoxia and normoxia were assessed using fluorescent microscopy from ten pairs of hemocytes in each group. In response to hypoxia, mitochondrial morphology was altered from filamentous to punctate structures, suggesting excessive mitochondrial fission. Following 24 h of hypoxia, there was significantly (*p* < 0.01) higher mitochondrial ROS production in the hypoxic hemocytes than in the control normoxia hemocytes (Fig. 1, *B*–*D*). To gain insights into the promotion of ferroptosis by hypoxia, we evaluated the impact of hypoxia on mitochondrial function. Relative to that in the normoxia group, the mitochondrial membrane potential was lower (*p* < 0.05) in hemocytes from prawns treated for 24 h with hypoxia (Fig. 1, *E* and *F*), suggesting increased mitochondrial dysfunction. Moreover, hypoxia led to a significant (*p* < 0.05) increase in lipid peroxide generation in hemocytes, as quantified using C11-BODIPY (Fig. 1*G*). Furthermore, a significant (*p* < 0.01) increase in the Fe^2+^ content, the formation of malondialdehyde (MDA), and the GSH content were observed in hemocytes following hypoxia exposure compared with those in the normoxia group (Fig. 1, *H*–*K*). Glutathione peroxidase 4 (GPX4) is a major defensive regulator against ferroptosis (27, 28). The protein level of GPX4 was significantly (*p* < 0.05) decreased in prawn hemocytes subjected to hypoxia for 24 h compared with those in the normoxia group, according to the Western blotting results (Fig. 1, *L* and *M*). Ferrostatin-1, as an effective ferroptosis inhibitor, rescued hypoxia-induced cell death and mitochondrial dysfunction (Fig. S1). Together, these results suggested that hypoxia has an important role in ferroptosis.

### Drp1 mitochondrial translocation is induced by hypoxia in prawn hemocytes *in vivo*

**Figure 2:**
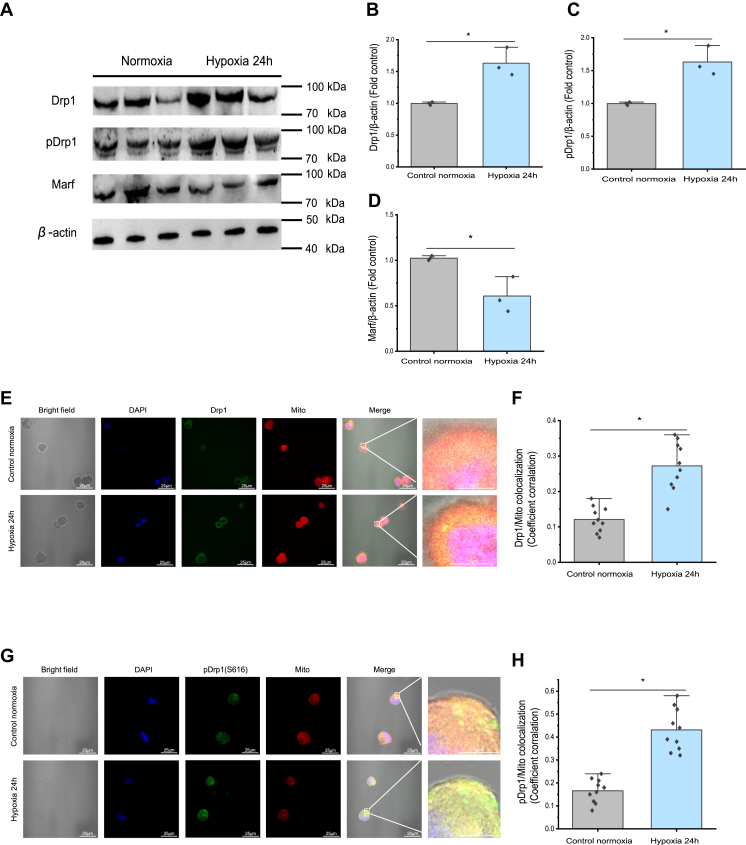
**Hypoxia-induced Drp1 protein expression and mitochondrial translocation in hemocytes of prawns *in vivo*.** The nucleotide sequence and the encoded amino acid sequence of prawn Drp1, the conserved GTPase binding domains, and a phylogenetic tree made using Drp1 protein sequences from the indicated species (see Table S2 for their accession numbers) are shown in Figs. S1–S3. *A*–*D*, representative images showing significant increases in the level of total Drp1 and pS616 Drp1, but significant decreases in the level of Marf, as determined using Western blotting (normalized to β-actin levels). ImageJ quantification of the protein bands shown in (*A*). *E*–*H*, increased localization of Drp1 and pS616 Drp1 in the mitochondria of hemocytes from prawns under hypoxia for 24 h, as assessed using immunofluorescence microscopy. Data appear as the mean ± SD (n = 10). ∗*p* < 0.05 (two-tailed unpaired Student’s *t* test) for the normoxia *versus* hypoxia for 24 h. Drp1, dynamin-related protein 1; Marf, mitochondrial assembly regulatory factor.

The prawn *Drp1* complementary DNA (cDNA) sequence encodes a protein of 748 amino acid residues (Fig. S2), which contains conserved predicted transmembrane regions (Fig. S3). The organization of the Drp1 domains resembles that of its human and mouse counterparts. Phylogenetic analysis of Drp1 proteins revealed two major branches: invertebrates (crustaceans, including prawn Drp1) and vertebrates (mammals and fish), which correspond to traditional zoological systematics (Fig. S4). The protein levels of total Drp1 and Drp1 phosphorylated at S616 increased significantly (*p* < 0.05) in prawn hemocytes subjected to hypoxia for 24 h compared with those in the normoxia group, according to the Western blotting results (Fig. 2, *A*–*C*). By contrast, the level of Marf (the analog of mammal MFN2), as a mitochondrial fusion protein from invertebrates, was significantly (*p* < 0.05) reduced in the hypoxia-treated hemocytes (Fig. 2*D*). In hypoxia-induced hemocytes, we observed mitochondrial colocalization of total Drp1 and pS616Drp1 (Fig. 2, *E*–*H*), as shown by immunofluorescence, suggesting enhanced Drp1 mitochondrial translocation in 24 h hypoxia-treated prawn hemocytes. These results indicated disruption in mitochondrial fusion and fission-related protein expression in hypoxia-induced hemocytes. Therefore, our subsequent experiments focused on the function of proteins related to mitochondrial dynamics under hypoxia.

### Overexpression of *Marf* attenuates mitochondrial dysfunction in the insect S2 cell under hypoxia *in vitro*

**Figure 3:**
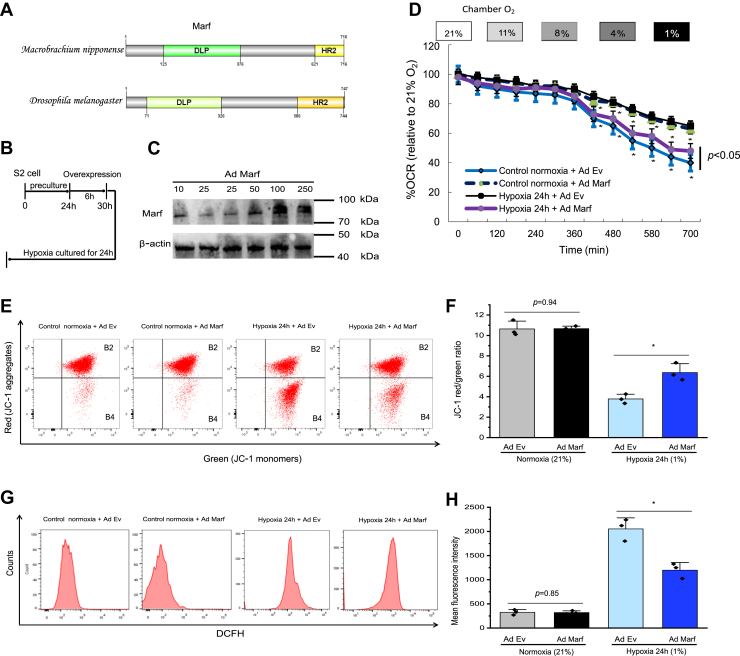
**Marf overexpression restored mitochondrial function in hypoxia-treated insect S2 cells *in vitro*.***A*, Marf protein domain organization from oriental river prawn (*Macrobrachium nipponense*) and fruit fly (*Drosophila melanogaster*). Conserved GTPase binding domains are shown. *B*, experimental scheme of the hypoxia-induced insect S2 cell experiment. *C*, representative Western blotting images are shown. Increasing Marf protein levels achieved using an adenoviral construct. *D*, the cellular OCR of S2 cells under hypoxia was plotted as percent of the OCR in 21% O_2_ (*y*-axis) *versus* time (*x*-axis) (n = 6). *E* and *F*, the mitochondrial membrane potential was detected using the sensitive dye JC-1. Flow cytometry data showing that Marf overexpression rescued the decreasing mitochondrial membrane potential in S2 cells induced by hypoxia. *G* and *H*, flow cytometry data showing that Marf overexpression attenuated the generation of cellular ROS in S2 cells induced by hypoxia. Data appear as the mean ± SD (n = 3). ∗*p* < 0.05; ∗∗*p* < 0.01 (two-tailed unpaired Student’s *t* test) for the control + adenoviral EV *versus* hypoxia or transduced Marf. EV, empty vector; Marf, mitochondrial assembly regulatory factor; OCR, oxygen consumption rate; ROS, reactive oxygen species.

Given that we have found that hypoxia decreased Marf protein levels, we evaluated Marf conserved domain conservation between *Drosophila melanogaster* and *Macrobrachium nipponense, as shown* (Fig. 3*A*). Using an adenoviral expression vector to increase *Marf* expression in insect S2 cells resulted in an increased mitochondrial membrane potential and mitochondrial oxygen consumption rate in hemocytes in response to hypoxia (Fig. 3, *B*–*D*), indicative of increased mitochondrial function. *Marf* overexpression rescued the hypoxia-inhibited mitochondrial membrane potential (Fig. 3, *E* and *F*). Furthermore, *Marf* overexpression decreased the hypoxia-induced cellular ROS production (Fig. 3, *G* and *H*). These results supported the conclusion that the disruption in mitochondrial fusion and fission balance in hemocytes underlies the observed mitochondrial dysfunction.

### Drp1's role in hemocyte function under hypoxic conditions *in vitro*

**Figure 4:**
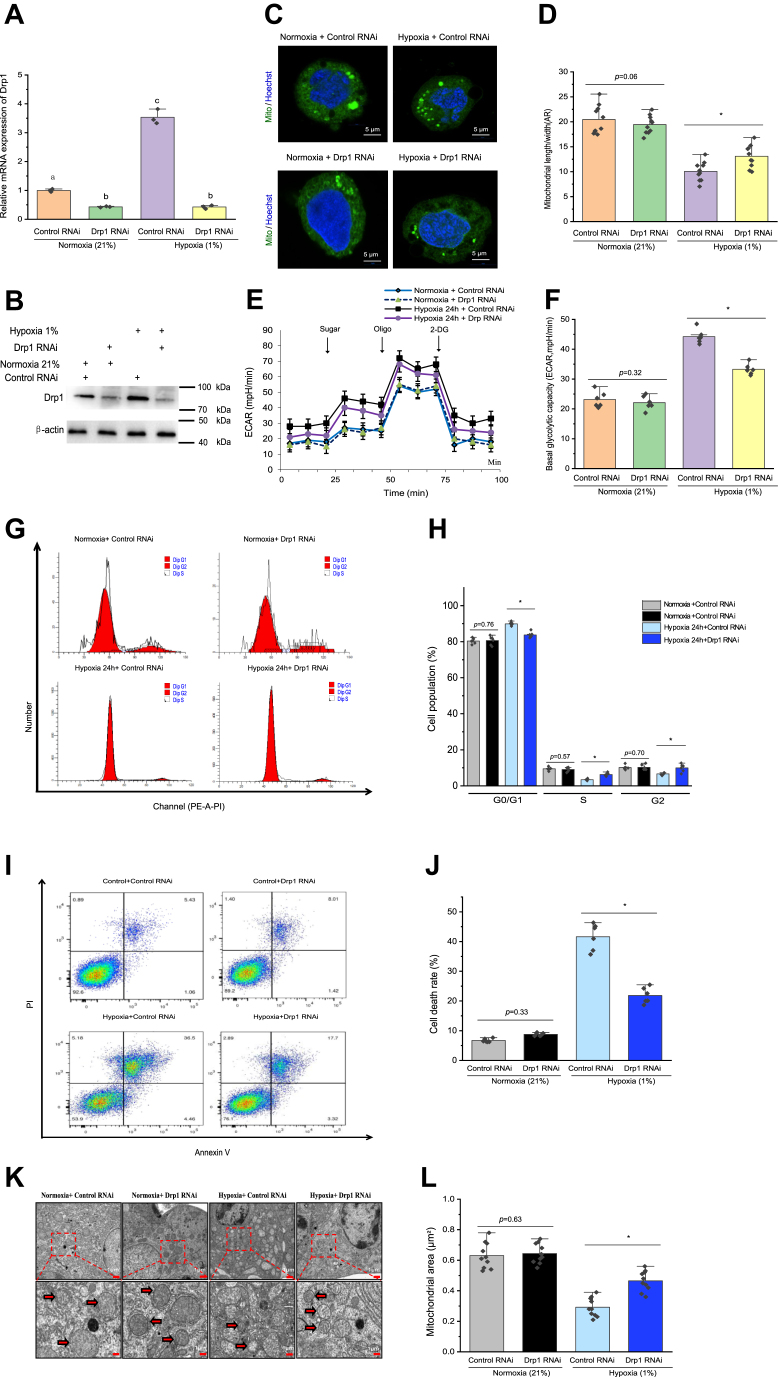
**Attenuating mitochondrial fission *via Drp1* knockdown reduces cellular dysfunction and apoptosis in hypoxic prawn hemocytes *in vitro*.***A*, *Drp1* mRNA expression in *Drp1* gene-silenced hemocytes and control hemocytes expressing a nontargeting siRNA, which were subjected to hypoxia or normoxia for 24 h. Shown are the means ± SD (n = 3). Three independent repeats were performed with different lowercase letters indicating the significance (one-way ANOVA). *B*, Western blotting confirmation of *Drp1* knockdown in hemocytes. *C* and *D*, *Drp1* knockdown inhibited the effect of hypoxia on mitochondrial fission. Data appear as the mean ± SD (n = 10). ∗*p* < 0.05, control siRNA sample *versus Drp1* knockdown treated hemocytes. *E* and *F*, *Drp1* knockdown attenuated the effect of hypoxia on cellular glycolysis, as evidenced by decreases in basal glycolytic capacity. *G*, effects on the cell cycle of hypoxia or *Drp1* gene silencing in hemocytes, as assessed using flow cytometry. *H*, summary data showing that *Drp1* silencing attenuated hypoxia's inhibitory effect on the hemocyte cell cycle. The error bars indicate the mean ± SD (n = 6). *I*, flow cytometry detection of the effect of *Drp1* silencing or hypoxia on cell apoptosis. *J*, summary data indicating that *Drp1* silencing abrogated hypoxia's induction of hemocyte apoptosis. The error bars indicate the mean ± SD (n = 6). *K*, transmission electron microscopy observations of mitochondrial ultrastructure in hemocytes. *L*, quantitative analyses of the mitochondrial area. Each dot represents the average length of mitochondria in one cell (n = 10). ∗*p* < 0.05 (two-tailed unpaired Student’s *t* test); the sample in the control siRNA group *versus* the sample in the *Drp1* siRNAs. Drp1, dynamin-related protein 1.

First, hemocyte *Drp1* was knocked down to ascertain whether Drp1 regulates mitochondrial fission under hypoxic conditions (Fig. 4*A*). After knockdown of *Drp1*, hypoxia was unable to induce an increase in the Drp1 protein content (Fig. 4*B*). *Drp1* knockdown also abrogated the hypoxia-induced increase in mitochondrial fission (Fig. 4, *C* and *D*). Next, we confirmed that knockdown of *Drp1* influenced the hypoxia-mediated alterations in the hemocytes' glycolytic capacity (Fig. 4, *E* and *F*). Compared with that in the control cells, the hemocyte cell cycle was inhibited by hypoxia, as assessed using flow cytometry analysis, and knockdown of *Drp1* significantly reversed this decrease *in vitro* (*p* < 0.01) (Fig. 4, *G* and *H*). In detail, following knockdown of *Drp1*, hemocytes entered the S phase from the G0/G1 phase more rapidly, but there was a significant increase in the duration of the G2 phase (*p* < 0.05). Moreover, relative to that in the scrambled control group, knockdown of *Drp1* in hemocytes significantly reduced the extent of hypoxia-mediated apoptosis (*p* < 0.05) under hypoxic conditions, according to flow cytometry analysis (Fig. 4, *I* and *J*). Moreover, knockdown of *Drp1* alleviated hypoxia-induced mitochondrial damage. Compared with that in the control normoxia hemocytes, transmission electron microscopy (TEM) observation revealed a reduction in size and enhanced loss of cristae in the mitochondria of hemocytes treated with hypoxia for 24 h, and knockdown of *Drp1* inhibited this decrease *in vitro* (Fig. 4*K*). In addition, knockdown of *Drp1* in hemocytes *in vitro* significantly reduced the extent of hypoxia-enhanced lipid peroxide generation and MDA content, which represent ferroptosis markers (Fig. S5). Drp1 gene silencing abrogated hypoxia's significant induction of shrunken mitochondria, which represents another morphological feature of ferroptosis (Fig. 4*L*).

### Yeast two-hybrid assay identification of the interaction between Drp1 and MTP18

**Figure 5:**
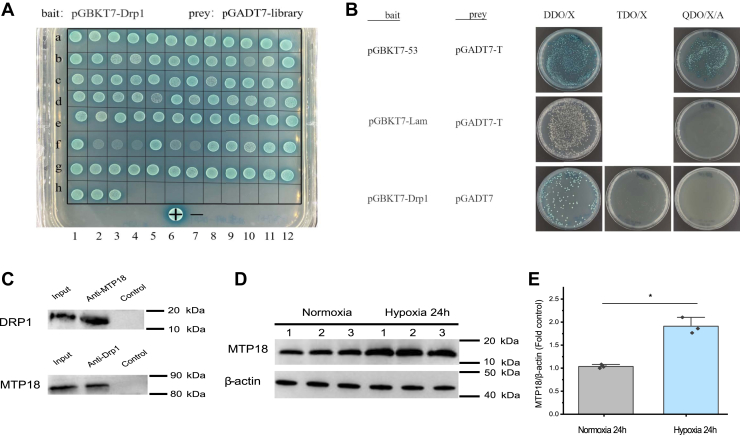
**MTP18 and Drp1 interact.***A* and *B*, proteins that interact with Drp1 identified using an Y2H screen. A Gateway AD library constructed using cDNA from prawn hemocytes, hepatopancreas, and gills was screened using Drp1 as the bait. Y2H confirmed six candidate Drp1 interacting proteins. The full-length *Drp1* cDNA was cloned into vector pGBKT7 and then transformed into yeast cells. +: pGBKT7–53 and pGADT7-T were transformed at the same time as positive controls; −: pGBKT7-Lam and pGADT7-T were transformed at the same time as negative controls. *C* and *D*, determination of the interaction between Drp1 and native MTP18 in the hemolymph of prawns *in vivo*. *Upper panel*, lane 1, Western blotting detection of Drp1 among prawn hemocyte total proteins. Lane 2, the anti-MTP18 antibody was incubated with hemocytes and then retrieved using Protein A/G beads. Western blotting was then used to detect bound proteins using anti-Drp1 antibodies. Lane 3, control (no specific bands). *Lower panel*, lane 1, MTP18 levels among total hemocyte proteins. Lane 2, the anti-Drp1 antibody was incubated with hemocyte proteins and then isolated with Protein A/G beads. Western blotting then detected the bound protein using anti-MTP18 antibodies. Lane 3, control (no specific bands). *E* and *F*, Western blotting showing that hypoxia increased the total MTP18 level. ImageJ quantification of the protein bands shown in (*E*). Data appear as the mean ± SD (n = 3). ∗*p* < 0.05 (two-tailed unpaired Student’s *t* test), control normoxia *versus* hypoxia for 24 h. cDNA, complementary DNA; Drp1, dynamin-related protein 1; MTP18, mitochondrial fission process protein 1; Y2H, yeast two-hybrid.

We used Drp1 as the bait sequence in a yeast two-hybrid (Y2H) assay to determine its potential interaction partners (Fig. 5*A*). This screen identified 87 possible prey proteins, among which 35 were deemed negative because they grew weakly on quadruple dropout supplements (QDO) plates. We sequenced the remaining clones (Table S1), among which clone d02, encoding a MTP18 homolog, attracted our attention, because MTP18 as a mitochondrial fission protein might help to recruit Drp1 proteins to the mitochondrial membrane (29). Pairwise analysis of full-length MTP18 and Drp1 supported their specific interaction (Fig. 5*B*). Co-immunoprecipitation demonstrated that Drp1 interacted with native hemocyte MTP18 *in vivo*, thus confirming the MTP18-Drp1 interaction, which possibly forms a complex that functions in mitochondrial fission (Fig. 5*C*). Furthermore, the profile of MTP18 protein expression was similar to that of Drp1 in 24-h hypoxia-treated prawn hemocytes (Fig. 5, *D* and *E*). The full role of MTP18 in the crustacean’s hemocyte remains to be determined.

### MTP18 expression is preferentially induced by HIF-1**α** during hypoxia

**Figure 6:**
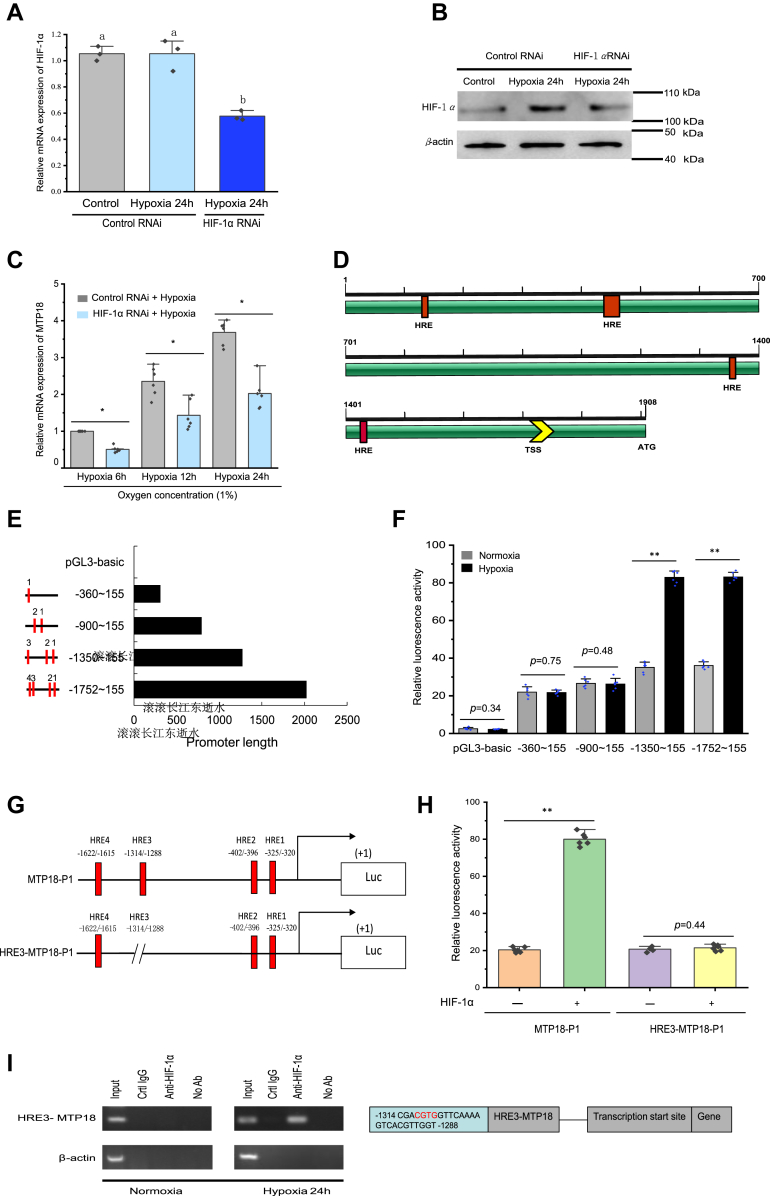
**MTP18 is preferentially induced *via* an HIF-1α binding site under hypoxic conditions.***A*, the effects of RNAi on HIF-1α. An HIF-1α siRNA (siHIF-1α) was designed to knockdown *HIF1a* expression, and *HIF1a* expression in prawn hemocytes was determined at 24 h post hypoxia with siHIF-1α using real time qRT-PCR (with siGFP as a control). Shown are the means ± SD (n = 3). Three independent repeats were performed with different lowercase letters indicating the significance (one-way ANOVA). *B*, hemocytes were knocked down for HIF-1α, which was confirmed using immunoblotting. *C*, the expression of *MTP18* mRNA under normoxia and hypoxia conditions. *D*, diagram of the *MTP18* promoter. Predicted TF binding sites are *underlined* and were identified using the TRANSFAC database (http://gene-regulation.com/) annotations. Fig. S5 shows the 5′ flanking region sequence (promoter) of prawn *MTP18*. *E*, schematic of the promoter-luciferase reporter constructs. *F*, the relative luciferase activity of each construct in transiently transfected insect S2 cells. Data appear as the fold induction compared with that of the empty vector. Data appear as the mean ± SD (n = 6), ∗*p* < 0.05 and ∗∗*p* < 0.01 (two-tailed unpaired Student’s *t* test). *G*, diagram of the mutated *MTP18* promoters. ΔHRE3-MTP18-P1 represents the MTP18 promoter mutated at putative HRE3. *H*, relative luciferase expression levels in insect S2 cells containing the promoter constructs and a plasmid expressing HIF-1α. Data appear as the means ± SD (n = 6), ∗∗*p* < 0.01 (two-tailed unpaired Student’s *t* test). *I*, HIF-1α binds to HRE3 in the *MTP18* promoter under hypoxic conditions in prawn hemocytes, as determined by ChIP assays. ChIP, chromatin immunoprecipitation; HIF-1α, hypoxia-inducible factor 1 alpha; HRE3, hypoxia response element 3; MTP18, mitochondrial fission process protein 1; real time qRT-PCR, quantitative real-time reverse transcription PCR; TF, transcription factor.

Next, a specific siRNA targeting the transcripts of *HIF1a* was designed to silence *HIF1a* expression. Successful knockdown of *Hif1a* was confirmed using quantitative real-time RT-PCR (real time qRT-PCR) (Fig. 5*A*). Subsequently, *HIF1a* knockdown suppressed the hypoxia-induced increase in the HIF-1α protein level (Fig. 5*B*). We cloned the full-length prawn *MTP18* cDNA from hemocytes (Fig. S6). Characterization of *MTP18* gene sequence in prawn is shown in Fig. S7. The results of real time qRT-PCR analysis showed that prawn hemocyte expression of *MTP18* mRNA was induced by hypoxia (Fig. 6*C*). Genome walking was then used to assess the *MTP18* gene 5′-flanking sequence (Fig. S8). Multiple HREs were predicted in this region (Fig. 6*D*). Next, these HREs were cloned and placed 5′ to a luciferase reporter gene to test their function in hypoxia-associated gene regulation (Fig. 6*E*). These constructs (pGL3-MTP18) were transfected into insect S2 cells, followed by hypoxia or normoxia treatment for 24 h. Under hypoxic conditions, in comparison with that of the first two HREs in the promoter (−900 to 155), the luciferase activity in the cells harboring the first three HREs (−1350 to 155) was significantly (*p* < 0.01) increased. This suggested hypoxia-mediated transcriptional activation acts *via* the third HRE in the *MTP18* promoter (−1350 to −900) (Fig. 6*F*). We then deleted 26 bp (−1288 to −1314) from HRE3 to form HRE3-MTP18-P1 (Fig. 6*G*), which was used for functional analysis of the HRE. Following transient transfection of HRE3-MTP18-P1, there was a significant (*p* < 0.01) decrease in HIF-1α-induced *MTP18* promoter activation following deletion of the presumed HRE3 site (Fig. 6*H*). In addition, chromatin immunoprecipitation (ChIP) assays showed that HRE3 is directly involved in hypoxia-induced *MTP18* transcriptional activation (Fig. 6*I*). These findings indicated that HIF-1α-induced *MTP18* promoter activation involves HRE3.

### The *in vivo* participation of MTP18 in mitochondrial fission and ferroptosis during hypoxia

**Figure 7:**
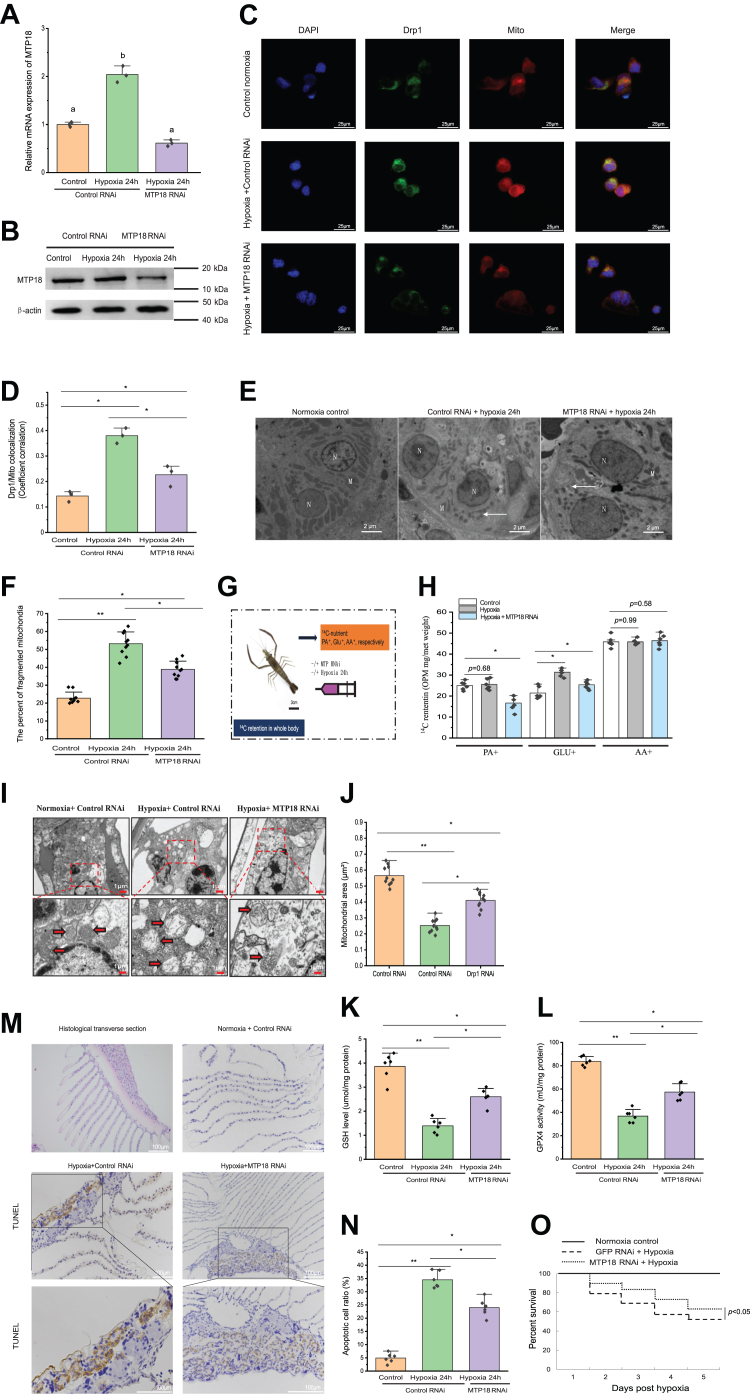
***MTP18* knockdown decreases *Drp1* expression levels to reverse mitochondrial fission and reduce cellular death in prawns treated with hypoxia *in vivo*.***A*, prawn hemocyte expression of *MTP18* was determined at 24 h after hypoxia in prawns silenced for *MTP18* or treated with siGFP as a control, as measured using real time qRT-PCR. Shown are the means ± SD (n = 3). Three independent repeats were performed with different lowercase letters indicating the significance (one-way ANOVA). *B*, Western blotting confirmation of *MTP18* gene silencing. *C* and *D*, *MTP18* knockdown inhibited the effect of hypoxia-induced Drp1 expression on its colocalization in hemocytes. Data appear as the mean ± SD (n = 3), ∗*p* < 0.05 (two-tailed unpaired Student’s *t* test) normoxia *versus* hypoxia groups (with the *MTP18* or control siRNA). *E* and *F*, mitochondrial status in the hemocytes of prawns silenced for *MTP18 in vivo* and exposed to hypoxia for 24 h. The *upper panel* shows representative electron microscopy images and the *lower panel* shows the summary data. M: mitochondria, N: nucleus. *Arrows* point to mitochondrial fission. The error bars indicate the mean ± SD (n = 6). ∗*p* < 0.05; ∗∗*p* < 0.01 (two-tailed unpaired Student’s *t* test). *G*, schematic diagram of ^14^C-labeled nutrient tracking test in prawns injected with 10 μg of siMTP18 (with siGFP as a control) after 24 h. *H*, ^14^C-retention in the whole body at 24 h after the injection of PA+ or Glu+, or AA+ in siGFP and siMTP18 knockdown prawns (n = 6), ∗*p* < 0.05 (two-tailed unpaired Student’s *t* test). *I* and *J*, transmission electron microscopy observations of mitochondrial ultrastructure in gill cells. Quantitative analyses of the mitochondrial area. Each dot represents the average length of mitochondria in one cell (n = 10). *K* and *L*, the GSH levels and GPX4 activity in *MTP18*-silenced prawns under hypoxia. GPX4 activity was analyzed using a GPX4 assay kit. Prawns were each injected with 10 μg of siMTP18 (with siGFP as a control), and GSH levels and GPX4 activity in the prawn’s gill tissue were determined 24 h later. *M* and *N*, knockdown of MTP18 rescued hypoxia-induced apoptosis in adult prawn gills according to TUNEL staining of the gills of *MTP18*-silenced prawns in response to hypoxia. Apoptotic cells are colored *brown*. *O*, the prawn survival rate was decreased by knockdown of *MTP18*. ∗*p* < 0.05; ∗∗*p* < 0.01 (two-tailed unpaired Student’s *t* test) in the normoxia *versus* hypoxia groups (with the *MTP18* or control siRNA). Drp1, dynamin-related protein 1; GPX4, glutathione peroxidase 4; MTP18, mitochondrial fission process protein 1; real time qRT-PCR, quantitative real-time reverse transcription PCR.

Fission is regulated by the mitochondrial membrane protein MTP18; however, whether its role is essential or redundant is unknown in invertebrates. To assess the role of MTP18 in Drp1 mitochondrial translocation *in vivo*, we knocked down *MTP18* in prawns, as confirmed using real time qRT-PCR (Fig. 7*A*). *MTP18* knockdown decreased the hypoxia-mediated enhancement of MTP18 protein abundance (Fig. 7*B*). Moreover, in comparison with that in prawns injected with the scrambled control siRNA, the *MTP18* knockdown prawns showed significantly (*p* < 0.05) decreased Drp1 mitochondrial translocation under hypoxia, as assessed using laser confocal scanning microscopy (Fig. 7, *C* and *D*). Then, TEM was used to count the number of mitochondrial fragments in adult prawns in the two groups under hypoxia. Thus, hypoxia-induced mitochondrial fission was arrested by *MTP18* knockdown (Fig. 7, *E* and *F*). To assess the role of MTP18 in the overall nutrient metabolism regulation *in vivo*, the use of [1-^14^C]-palmitic acid (PA+), D-[1-^14^C]-Glu (Glu+), and L-[^14^C(U)]-amino acid (AA+) were tracked after their individual intraperitoneal injection (Fig. 7*G*). The radioactivity retained in the bodies of prawns injected with PA∗ was significantly (*p* < 0.05) lower in the *MTP18* knockdown group, whereas the radioactivity retained after Glu∗ injection was significantly (*p* < 0.05) higher than that in the control normoxia group (Fig. 7*H*), suggesting a key role of MTP18 in glycolysis regulation. Lastly, compared with that in the control normoxia gill cells, TEM revealed a reduction in size and enhanced loss of cristae in the mitochondria of gill cells treated with hypoxia for 24 h (Fig. 7, *I* and *J*), and knockdown of *MTP18* obviously inhibited ferroptosis. In hypoxia-induced gill tissue, the prawns knocked down for *MTP18* had significantly (*p* < 0.05) reduced levels of GSH and showed significantly (*p* < 0.05) decreased GPX4 enzyme activity (Fig. 7, *K* and *L*), suggesting that *MTP18* knockdown not only decreased mitochondrial fission, but also inhibited oxidative damage to gill tissue. Therefore, we counted the number of apoptotic gill cells in *MTP18* knockdown adult prawns and in the scrambled control siRNA group under hypoxia using TUNEL assays. The gills of the *MTP18* knockdown prawns contained significantly (*p* < 0.05) fewer apoptotic cells than that in the control siRNA group after 24 h of hypoxia (Fig. 7, *M* and *N*). Indeed, under hypoxia, the prawns knocked down for *MTP18* had a markedly augmented survival rate compared with that of the control group (Fig. 7*O*).

## Discussion

In invertebrate research, a primary mechanism underpinning adaptation to hypoxia is HIF-1 signaling, which induces adaptive gene expression (30, 31, 32). An earlier study revealed the functional conservation of the HIF-1 pathway in fruit flies (*D. melanogaster*), which was also supported by HIF-1 promotion of hypoxia adaptation in the nematode *Caenorhabditis elegans* (33, 34), implying its long-standing evolutionary inception. Recently, research has revealed that the glycolytic pathway and mitochondria, as ATP producers, are the important HIF-dependent adaptations (35). All eukaryotic cells contain mitochondria, which have a broad range of vital functions, such as energy production and respiration. Importantly, mitochondrial function is affected by changes in the activities of proteins associated with mitochondrial dynamics. Previous studies using fruit flies showed that *in vivo*, imbalanced mitochondrial homeostasis resulting from the knockdown of *Drp1*, *Opa1*, and *Marf*, respectively, caused mitochondria fragmentation and a major deficit in oxidative phosphorylation (36, 37, 38, 39). ATP synthesis and production mainly occur *via* oxidative glucose metabolism through respiration, suggesting that it is difficult to supply enough energy to insects when their mitochondria are dysfunctional.

The *Drp1* gene has been widely confirmed to exist in fish, nematodes, and prawns (40, 41, 42). Herein, the observation of the key functions of Drp1 in prawns deepens our understanding of the effects of hypoxia stress on crustaceans. Furthermore, our experimental data suggested that HIF-1α-mediated upregulation of MTP18 participates in hypoxia-induced Drp1 mitochondrial translocation. In summary, the hypoxia-increased excess mitochondrial fission is associated with prawn mitochondrial dysfunction through the HIF-1α- MTP18/Drp1 axis in hemocytes. The vast majority of invertebrates have an open circulatory system, and their body fluids (hemolymph) also leave the heart through arteries, but do not flow into capillaries. The change to a glycolytic phenotype could be particularly useful for hemocytes in which oxygen availability should decline rapidly but ATP production needs to be maintained for the highly energetic process of hemolymph flow.

In recent years, the relationship between mitochondria and ferroptosis has received increased attention. The traditional view is that ferroptosis is triggered by the accumulation of ROS because mitochondria are the major sources and primary targets of ROS (43, 44). A previous study showed that hypoxia inhibited mitochondrial complex I in melanoma cells, which triggered mitochondrial dysfunction, leading to ferroptosis (45). This was similar to the results of the present study, in which hypoxic stress might trigger ferroptosis by causing mitochondrial dysfunction. Another reasonable explanation was that hypoxia stimulates HIF-1α transcription in human cells, which enhances fatty acid uptake and lipid storage, and ultimately the cells undergo excessive lipid peroxides and ferroptosis (46, 47).

To date, mitochondrial fission and fusion processes in invertebrates have been determined mainly in *D. melanogaster* (48). However, in nonmodel invertebrates, the pathway of mitochondrial dynamics is poorly explored. In the draft genome sequence assembly of *M. nipponense*, we noted a number of genes encoding conserved dynamin-like GTPases, such as Marf and Drp1. To produce cellular energy, the functional homeostasis of mitochondria is maintained by successive rounds of fission and fusion (49, 50). Thus, we initially sought to determine whether an imbalance of mitochondrial dynamics was induced by hypoxia. Interestingly, in the hypoxia group, besides Drp1 activation, the expression levels of Marf were decreased. Thus, under hypoxia, hemocytes likely experience excess mitochondrial fission. Our findings indicated that *Marf* overexpression rescued hypoxia-induced mitochondrial dysfunction. This was supported by the observation that hypoxia decreased cardiomyocyte viability and disrupted mitochondrial fusion, and both effects were promoted by the knockdown of a mitochondrial fusion protein (51). Our results also confirmed that Drp1 is commonly located in the cytoplasm and mitochondria under basal conditions, but upon hypoxia-mediated activation, more Drp1 is recruited to the mitochondrial surface for mitochondrial fission, similar to earlier reports in mammals (52, 53). These results imply that upregulation of Drp1 is vital for hemocytes to respond to hypoxia stress, subsequently resulting in mitochondrial fission. For mitochondrial fission, Drp1 forms helical oligomers on the outer mitochondrial membrane, leading to membrane constriction and fragmentation (54, 55). The results of the present study provide support for the hypothesis that activation of Drp1 *via* S616 phosphorylation augments mitochondrial fission (56, 57, 58). Thus, Drp1 phosphorylation is an essential regulator of its activity in crustaceans. In addition, inhibition of Drp1 reduced mitochondrial fission. The *Drp1* knockdown-mediated decrease in ROS production and apoptosis might enhance cell survival *in vivo* (59, 60, 61, 62).

In the life cycle of mitochondria, mitochondrial biogenesis and apoptotic clearance of dysfunctional mitochondria are both enabled by fission. Current models of the regulation of mitochondrial fission reveal that excessive mitochondrial fission induced by hypoxia follows a decrease in membrane potential and augmented ROS levels, which occur long before ferroptosis (63, 64). We found that the hypoxia-promoted excessive mitochondrial fragmentation leads to mitochondrial dysfunction. Therefore, hypoxia might induce ferroptosis through Drp1-mediated mitochondrial fission. To verify our hypothesis, Drp1 RNAi was applied in hypoxic stress-treated prawns. Our results showed that *Drp1* knockdown inhibited ferroptosis, accompanied by mitochondrial cristae remodeling. These findings supported the importance of Drp1-mediated mitochondrial fission in hypoxic stress-induced ferroptosis, which was consistent with a recent report in which Drp1 depletion protected against ferroptotic cell death by preserving redox homeostasis (65).

We identified a new mechanism by which HIF-1α induces MTP18 expression under hypoxia, and observed that glycolysis events are enhanced in the hepatopancreas and gills of prawns under hypoxia (66). Consequently, the association between the activation of glycolysis/mitochondrial dysfunction and the decrease in the mitochondrial membrane potential caused by hypoxia was confirmed. Herein, increased HIF-1α levels were observed to accompany MTP18 upregulation. Drp1 is recruited to the mitochondria by MTP18 (67). *MTP18* deletion triggered mitochondrial hyperfusion in some types of cells (68). However, the impact of *MTP18* knockdown on the conserved pathway of crustacean mitochondrial fission has not been investigated. Correspondingly, we clearly demonstrated that *MTP18* knockdown abrogated the hypoxia-induced augmentation of the mitochondrial translocation of Drp1, suggesting a role for mitochondrial anchored recruiters. Consistently, *MTP18* knockdown in hemocytes reduced mitochondrial fission and glycolysis. Importantly, the *in vivo* knockdown of *MTP18* also increased the survival rate significantly in hypoxia-treated prawns, which was consistent with previous studies in which *MTP18* downregulation prevented the fragmentation of mitochondria, preserved mitochondrial respiration function, and protected mammalian cells from apoptosis (69, 70, 71).

The Drp1/MTP18 interaction was confirmed by Y2H assays and immunoprecipitation in hypoxia-induced prawn hemocytes. Previous studies proposed that MTP18 has a central role in the control of mitochondrial dynamics upstream of DRP1, which supports our findings (72, 73). Thus, we speculated that in crustaceans subjected to hypoxia, the excess mitochondrial fission induced by hypoxia might be mainly driven by the interaction between Drp1 and MTP18, rather than other mitochondrial fission regulators (*e.g.*, FIS1, MFF, MiD49, and MiD5), which has certain implications for the evolution of the fine-tuning of the hypoxic response. Our findings suggest that the impact of hypoxia is counteracted by Drp1/MTP18 in crustaceans; however, it is possible that other, as yet unidentified, Drp1 adaptors also contribute. Importantly, *MTP18* knockdown *in vivo* also significantly decreased hypoxia-mediated ferroptosis. Indeed, the decreased apoptosis in the gill tissue of *MTP18* knockdown prawns under hypoxia was related to the inhibition of excessive mitochondrial fission.

**Figure 8:**
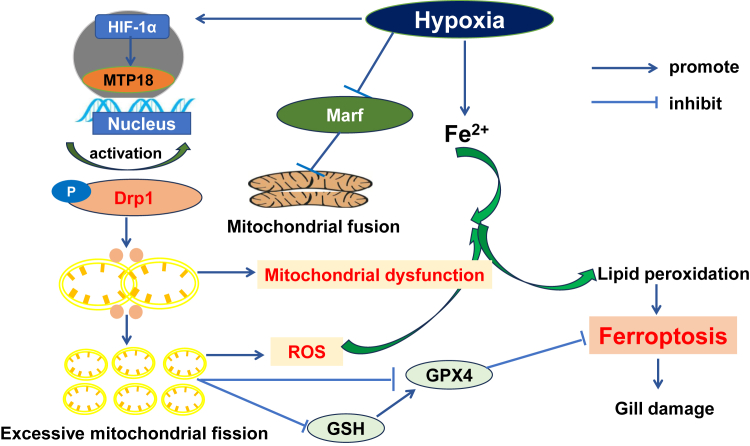
**Diagram of enhanced mitochondrial dysfunction-induced ferroptosis through activation of MTP18/Drp1 mediated by hypoxia.** Increased cellular mitochondrial fragmentation occurred *via* activation of MTP18/Drp1, mediated by hypoxia for 24 h, which lead to enhanced mitochondrial dysfunction and ferroptosis. Drp1, dynamin-related protein 1; GPX4, glutathione peroxidase 4; HIF-1α, hypoxia-inducible factor-1α; Marf, mitochondrial assembly regulatory factor; MTP18, mitochondrial fission process protein 1; ROS, reactive oxygen species.

In conclusion, this study improves our understanding of the mechanism by which hypoxia induces mitochondrial dysfunction and ferroptosis in prawn hemocytes (Fig. 8). This study revealed that MTP18 interacts with and recruits Drp1 to mitochondria, and this interaction functions in the hypoxia-induced excess mitochondrial fission in the hemocytes of invertebrates. These results provide new insights into the regulation of ferroptosis in invertebrates in response to hypoxia and shed light on the conserved evolution of MTP18/Drp1-mediated fission within the animal kingdom, possibly leading to the development of efficient strategies to maintain animal health under hypoxic stress.

## Experimental procedures

### Experimental prawns and culture of primary hemocytes

All the animal experiments were approved by the Committee on the Use of Live Animals in Teaching and Research of the Shanghai Ocean University. We purchased the healthy oriental river prawns from Donghai aquatic farm (Shanghai, China). The prawns were rapidly shipped to the laboratory at Shanghai Ocean University. Thoroughly aerated and filtered fresh water was used to culture the prawns, which were fed with a commercial formula diet once per day. The prawns underwent a 14-day acclimation period as follows: DO 6.5 ± 0.2 mg/l, pH 7.8 ± 0.1, and 24.5 ± 0.5 °C. For tissue distribution analysis, the hemolymph from 10 healthy prawns was extracted into an Eppendorf tube (1.5 ml), added with the same volume of anticoagulant solution, and centrifuged immediately at 1000*g* for 10 min. The pellet was retained. Previously published methods (74) were employed for hemocyte primary culture. Hemolymph-derived insect S2 cells were grown in *Drosophila* Schneider’s medium (Gibco) containing antibiotics and 10% fetal bovine serum. To induce hypoxia in the cells, we used a hypoxic workstation (Ruskinn Technology) in which the cells were maintained in a normoxic incubator (21% O_2_ and 5% CO_2_) or in a hypoxic incubator (1% O_2_, 5% CO_2_, and 94% N_2_). Cells between passages three and eight were used in all experiments.

### Hypoxia stress and the collection of samples

After acclimation, the control prawns were reared under normoxic conditions (DO = 6.5 ± 0.2 mg/l). In the hypoxia group, N_2_ gas was used to maintain a DO of 1.8 ± 0.1 mg/l for 24 h (75). Then, 120 male prawns (3.15 ± 0.52 g) were assigned randomly to six tanks (20 prawns per tank), followed by normoxia (control) treatment or hypoxia treatment for 24 h (with triplicate groups for each treatment). Sampled hemocytes were frozen immediately and stored at −80 °C for subsequent extraction of mRNA and protein. Identical experiments were carried out using a minimum of three biological repeats.

### RNA isolation, cDNA cloning, and bioinformatics

RNA extraction and cDNA synthesis were carried out using the methods detailed by Sun *et al.* (76). The mRNAs encoding prawn *Drp1* and *MTP18* were identified using the *M. nipponense* transcriptome database (SRP110812; National Center for Biotechnology Information). The *Drp1* and *MTP18* cDNAs were cloned according to the methods detailed by Sun *et al.* (75). After purification, sequencing of the PCR products was carried out, followed by ClustalW2 analysis (http://www.clustal.org/clustal2/). Functional protein domains in the encoded proteins were predicted using SMART version 4.0 (http://smart.embl-heidelberg.de/). The tertiary structures of the proteins were predicted using the SWISS-MODEL server on ExPASy (https://www.expasy.org) and visualized employing PyMOL software (https://www.pymol.org). The neighbor-joining method in MEGA4 (https://www.megasoftware.net/mega4/mega.html) was utilized to construct a phylogenetic tree. The corresponding accession numbers are shown in Table S2.

### Mitochondrial membrane potential detection using JC-1

The cellular mitochondrial membrane potential (MMP) was determined using a JC-1 (5,5′,6,6′-tetrachloro-1,1′,3,3′- tetraethylbenzimidazolylcarbocyanine iodide) Mitochondrial Membrane Potential Assay Kit (Cayman Chemical, Inc) following the vendor's instructions (77). The lipophilic, cationic dye JC-1 selectively enters the mitochondria, wherein, as the MMP increases, it undergoes a reversible change in color from green to red. Briefly, 1 μl (5 μM) of JC-1 was incubated with hemocytes (1 × 10^6^ cells/ml in cell suspension buffer) in the dark for 25 min with gentle agitation. The cells were then rinsed twice using fresh media, resuspended in 2 ml of medium, and added to a fluorimeter cuvette. An emission filter centered at 595 nm (25 nm bandwidth) was used to detect the peak excitation at 560 nm of the red fluorescence (J-aggregates). Excitation (485 nm) and emission (535 nm) were used to detect the green fluorescence of JC-1 monomers. An Olympus IX51 microscope (Olympus) equipped with a charge-coupled device camera (Hamamatsu Photonics) was used to capture the fluorescence images. The fluorescence intensity was calculated using ImageJ/Fiji software (https://imagej.net/imagej-wiki-static/Fiji).

### Analysis of mitochondrial ROS and TEM

Mitochondrial ROS were detected using a MitoSOX Red mitochondrial superoxide indicator (#M36008, Thermo Fisher Scientific). The Olympus IX51 microscope was employed to capture the fluorescent images (78). ImagePro Plus imaging software (https://www.ms-imaging.com/image-pro-plus-software) was employed to quantify the mean fluorescent intensities (to allow for variations in the number of cells). Mitotracker (30 nM, #M7512, Invitrogen) was used to label mitochondria in cells at 37 °C for 15 min; nuclei were stained using Hoechst 33342 (#R37605, Thermo Fisher Scientific). An Olympus confocal laser scanning microscope (Olympus America Inc) was then utilized to capture high-sensitivity and low-noise fluorescence images. ImageJ/Fiji (https://imagej.net/imagej-wiki-static/Fiji) software was employed to analyze the morphology of mitochondrial fission. Particle analysis was used to determine the long and wide axes of the mitochondria. The sizes of the mitochondria were analyzed by morphological measurements using TEM analysis software (https://www.gatan.com/products/tem-analysis) and ImageJ software. The characteristics of mitochondrial fragmentation were represented by calculating the aspect ratio (79, 80, 81). TEM (Hitachi H-7650) was used for ultrastructural analyses, as previously described (82). In the different samples, at least 100 individual mitochondria per group were used to quantify mitophagy. The extent of mitochondrial fragmentation was represented as the percentage of fragmented mitochondria among all the mitochondria in the sample.

### Flow cytometry analysis

Primary-cultured hemocytes or insect cells were exposed to hypoxia for 24 h. A ROS assay kit, including the oxidant-induced fluorescent probe 2′,7′-dichlorofluorescein-diacetate (Nanjing Jiancheng BIO, Inc) was used to determine the intracellular ROS concentrations in hemocytes. Then, the transfected hemocytes were gently washed three times with PBS and assessed using flow cytometry (CytoFLEX S; Beckman Coulter) at the Gap 1 (G1), synthesis (S), and Gap 2 (G2) phases of the cell cycle. An Annexin V-enhanced GFP/propidium iodide apoptosis detection kit (KeyGEN Biotech) was used to assess apoptosis, and the apoptotic cells were detected using a flow cytometer. Following gene silencing, hypoxia was induced in hemocytes for 24 h, followed by gentle washing with PBS three times. The gene-silenced hemocytes were then subjected to flow cytometry (CytoFLEX S) analysis for the G1, S, and G2 phases. Apoptosis was assayed using the Annexin V-EGFP/PI apoptosis detection kit, followed by flow cytometry detection of apoptotic cells. Six independent replicates were assessed in these experiments.

FeRhoNox-1 (Cat: HY-D1533; MedChemExpress), a commercial fluorescent probe that specifically binds Fe^2+^, was used to determine the intracellular Fe^2+^ content. The hemocytes in the different treatment groups were immobilized with 4% paraformaldehyde for 1 h and washed thrice with PBS. After the addition of 5 Μm FeRhoNox-1, the stained cells were placed in the dark for 20 min. Then, Fe^2+^ images were obtained using a fluorescence microscope. C11-BODIPY581/591 (Cat: D3861), a marker for lipid peroxidation obtained from Thermo Fisher Scientific, was used to assess lipid peroxidation. Hemocytes were incubated with 2 μM C11-BODIPY581/591 for 30 min at 37 °C in the absence of light. Flow cytometry measurements were then carried out according to a previously described method (83). The fluorescence intensity of each probe was quantified using the FlowJo software program (https://www.flowjo.com/).

### Luciferase reporter plasmid construction, transfection, and activity analysis

PCR with primers P1–P4 (Table S3) was used to serially truncate the prawn *MTP18* promoter. Transfection and the luciferase assays (under normoxic and hypoxic conditions for 24 h) were carried out according to previously described methods (84). The activity of firefly luciferase was normalized to the activity of Renilla luciferase (internal control), calculated using a Dual-Luciferase reporter assay system (Promega). The HRE motifs in the *MTP18* promoter were mutated using a QuikChange Site-Directed Mutagenesis Kit (Stratagene) with pairs of mutagenic primers, according to the supplier’s guidelines. The mutated HRE sites were verified using DNA sequencing.

### Quantitative real-time reverse transcription PCR

Total RNA extracted from prawn cells and tissues was reverse transcribed to cDNA (85). The cDNA was then quantified in the qPCR step of the real time qRT-PCR protocol using a SYBR Green kit (Takara) and the LightCycler system (Roche). The primers for amplification of the target genes and the β-actin encoding mRNA (internal control) are listed in Table S3. We created standard curves using serially diluted pure cDNA samples to estimate each primer's amplification efficiency, which ranged from 0.95 to 1.05. The 2^−ΔΔCT^ method (86) was used to determine the relative expression level of each target gene.

### Adenovirus-mediated expression and RNA interference

The *Marf* overexpression adenovirus and negative control vector were purchased from Shanghai Sangon Biotech Co, Ltd. Insect S2 cells were seeded in a 6-well plate at a concentration of 1 × 10^5^ cells/ml and cultured for 24 h. Then, 1 μl of different concentrations of the *Marf* overexpression adenovirus and control vector were added into each well and transfected for 6 h, as described previously (87). The culture medium was then replaced, and the cells were cultured in a hypoxic incubator for 48 h. Primers containing a T7 promoter (Table S3) and a T7 transcription kit (Fermentas) were employed to amplify fragments of the *Drp1* and *MTP18* cDNAs for use as templates to produce siRNAs. For *in vitro* RNAi, the siRNA was dissolved in RNase-free water and transfected into primary-cultured hemocytes using Lipofectamine 3000 (Thermo Fisher Scientific) at a final concentration of 10 nM. The control comprised a GFP siRNA (GenePharma Co). For *in vivo* RNAi, the dsRNA (2 μg/g) was injected into the hemolymph of each prawn. The efficiency of siRNA-mediated gene silencing was assessed using real time qRT-PCR, with dsGFP RNA as the control. After the RNAi assay was set up, hypoxia was induced in siRNA-transfected hemocytes or siRNA-transfected prawns for 24 h, as previously described (42).

### Western blotting and immunofluorescence staining

After hypoxia treatment, hemocyte cellular proteins were extracted, and their total levels were assessed using a Pierce bicinchoninic acid protein assay kit (Thermo Fisher Scientific). Then, equal amounts of protein (50 μg) were subjected to electrophoretic separation through 10% SDS-polyacrylamide gels, and then electrophoretically transferred onto a polyvinylidene fluoride membrane (Millipore) (88). Antibodies against HIF-1α (ab501608), Drp1 (ab184247), MTP18 (ab198217), and β-actin (ab8224) were used (all Abcam). No nonspecific signals were generated using these antibodies. The primary antibodies (1:500 diluted) were incubated with the polyvinylidene fluoride membranes, washed thrice using Tris-buffered saline, added with alkaline phosphatase-conjugated goat anti-rabbit immunoglobulin G (IgG) (1:10,000 in Tris-buffered saline), and incubated for 3 h; the unbound antibodies were then washed off. The internal control comprised β-actin. An enhanced chemiluminescence detection system was used to visualize the immunoreactive protein bands on the membranes, which were quantified using ImageJ software (Version 1.51).

For immunofluorescence staining, 4% paraformaldehyde in PBS was used to fix the hemocytes for 20 min, after which the cells were transverse sectioned and mounted on slides. The slides containing the cells were incubated with primary antibodies followed by labeled secondary antibodies. The Olympus confocal laser scanning microscope was used to observe and image the cells according to a previously detailed method (89). The images were processed and analyzed for colocalization correlation with the ImagePro Plus imaging software (https://www.ms-imaging.com/image-pro-plus-software) or ImageJ (NIH).

### Y2H assay

The Drp1-interacting proteins and pairwise protein-protein interactions were detected using a Y2H assay employing the Matchmaker Gold Yeast Two-Hybrid System (Clontech; 630495). A Gateway activation domain prey library of oriental river prawn was constructed by OE Biotech Company and was used to identify Drp1-interacting proteins. TRIzol (Invitrogen; 15596–026) was used to extract total RNA from the hemocytes, hepatopancreas, and gills of prawns after hypoxia for 24 h, which were mixed equally. OE Biotech then created a cDNA library *via* reverse transcribing the extracted total RNA to cDNA, followed by cloning into the pGADT7 vector, as previously described (90). The Matchmaker Two-Hybrid system (Clontech) was then used to carry out the Y2H screening as previously described. Protein-protein interactions were recognized in the clones that grew on QDO/A medium and the blue clones that grew on QDO/X/A medium. An Easy Yeast Plasmid Isolation Kit (Clontech) was used to extract the prey plasmids from the positive clones, which were then sequenced.

### Co-immunoprecipitation assay and ChIP assay

Hemocyte proteins (500 μl) were incubated with 3 μl of anti-prawn Drp1 or 3 μl of anti-prawn MTP18 antibodies, followed by slow overnight shaking at 4 °C. The samples were added with protein A/G magnetic beads for immunoprecipitation (Selleck) and incubated at 4 °C for 3 h to capture the antigen-antibody complexes, as previously described (91). The samples were then analyzed using Western blotting. Purified rabbit IgG was used as the control. ChIP was carried out using a ChIP assay kit (Millipore) following the manufacturer’s protocol. ChIP was performed for prawn hemocytes cultured under hypoxic or normoxic conditions using anti-HIF-1α antibodies. As a control for nonspecific genomic DNA binding, normal IgG was used. The DNA fragments pulled down using the anti-HIF-1α antibodies were subjected to conventional PCR (70).

### Mitochondrial bioenergetics

Glucose-containing XF base medium was used in the oxygen consumption rate analysis, following the manufacturer’s protocol (Seahorse Biosciences). The extracellular acidification rate was evaluated using the XF Cell Mito Stress Test Kit in combination with a XF24 Analyzer (both from Seahorse Biosciences) as described previously (92).

### Metabolic tracking test of ^14^C-labeled nutrients

After prawns were injected with 10 μg of siMTP18 (with siGFP as a control), 36 prawns (12 prawns for each treatment group) were used to perform the metabolic tracking of ^14^C-labeled nutrients, following a 24-h fasting period, with or without hypoxia treatment. The metabolic tracking test was determined by injecting [1-^14^C]-PA (PA∗) or D-[1-^14^C]-glucose (Glu∗) or L-[^14^C(U)]-amino acid mixture (AA∗) (PerkinElmer) on the base of the third pereiopod, according to previous studies (93). Hemocyte lactate and ATP levels were measured using a lactate content detection kit (Beijing Solarbio Science & Technology Co, Ltd) following the supplier’s guidelines.

### Assessment of GSH and MDA

Total glutathione in hemocyte lysates or tissue lysates were measured using a GSH detection kit (Cat: S0053; Beyotime) according to the manufacturer’s instructions. The MDA in hemocyte lysates was measured using an MDA detection kit (Cat: S0131S; Beyotime) according to the manufacturer’s instructions.

### Cell viability and TUNEL assay

Cell viability was evaluated using a cell counting kit 8 (CCK8) assay. For the CCK8 assay, 10 μl of CCK8 solution (#521942, Bio shark) was added to primary-cultured hemocytes that had been treated with hypoxia and ferrostatin-1, and incubated for 0.5 h. The absorbance at 450 nm was then measured. Prawn gill tissue cell apoptosis was assessed using TUNEL assays for samples from prawns subjected to RNAi and hypoxia for 24 h, as described previously (94). TUNEL-positive cells were counted under a fluorescence microscope.

### Prawn survival

Twenty prawns per group were assigned randomly to receive *MTP18* siRNA injection or *GFP* siRNA injection. After *MTP18* gene silencing, all prawns were cultured in three hypoxia tanks. Deceased prawns in each tank were enumerated daily to determine the survival rate.

### Statistical considerations

The statistical analyses were carried out using SPSS software (https://spssai.statistical-analysis.top/). Data are presented by the mean ± SD. Statistical comparisons were performed. The differences between two groups were tested using Student’s *t* test. Since the sample size is less than/equal to n = 6 for several of the experiments, the appropriate test would be the nonparametric two-tailed Mann–Whitney test. For a sample size greater than 6, a normality test before utilizing the Student’s *t* test was used. For the comparison between more than two groups, if the sample size was less than 6, and normality is not assumed, the correct nonparametric test to be used should be Kruskal–Wallis analysis of variance (ANOVA) followed by *post hoc* tests. A *p* value < 0.05 or a *p* value < 0.01 was considered statistically significant.

## Data availability

All data are contained within the article.

## Supporting information

This article contains supporting information.

## Conflict of interest

The authors declare that they have no conflicts of interest with the contents of this article.
