# Supplementary material for: Hypoxia induces ferroptotic cell death mediated by activation of the inner mitochondrial membrane fission protein MTP18/Drp1 in invertebrates
Source: J Biol Chem. 2025 Feb 18;301(3):108326. doi: 10.1016/j.jbc.2025.108326 (PMC11957787; doi:10.1016/j.jbc.2025.108326)
Supplement: Supporting information [file mmc1.docx]

**Supplementary Tables**

**Table S1.** List of the potential interaction partners of Drp1

| Number | Candidate gene | Definition |
| --- | --- | --- |
| 1 | SZ-AD-SSM-Drp1-d01 | Bax [*Macrobrachium nipponense*] |
| 2 | SZ-AD-SSM-Drp1-c02 | Cathepsin L1 [*Macrobrachium nipponense*] |
| 3 | SZ-AD-SSM-Drp1-d02 | MTP18 [*Macrobrachium nipponense*] |
| 4 | SZ-AD-SSM-Drp1-d04 | Cyclooxygenase-2 [*Macrobrachium nipponense*] |
| 5 | SZ-AD-SSM-Drp1-d08 | FUNDC1 [*Macrobrachium nipponense*] |
| 6 | SZ-AD-SSM-Drp1-d09 | No significant similarity found |

**Table S2.** DRP1 and MTP18 amino acid sequences from different species

| Gene | species name | GenBank accession No |
| --- | --- | --- |
| Drp1 | *Macrobrachium nipponense* | MZ_823353.1 |
|  | *Litopenaeus vannamei* | XP_027210109.1 |
|  | *Cherax quadricarinatus* | AJW83245.1 |
|  | *Procambarus clarkii* | XP_ 045597352.1 |
|  | *Penaeus japonicus* | XP_ 042893203.1 |
|  | *Portunus trituberculatus* | XP_ 045127886.1 |
|  | *Homo sapiens* | NP_001265393.1 |
|  | *Mus musculus* | NP_001392182.1 |
|  | *Rattus norvegicus* | XP_038943821.1 |
|  | *Callithrix jacchus* | XP_003733777.1 |
|  | *Drosophila busckii* | ALC40131.1 |
|  | *Diprion similis* | XP_046747341.1 |
|  | *Cryptotermes secundus* | PNF28899.1 |
|  | *Apis cerana* | XP_016921510.1 |
|  | *Hippoglossus hippoglossus* | XP_034444497.1 |
|  | *Danio rerio* | NP_957216.1 |
|  | *Cheilinus undulatus* | XP_041652479.1 |
|  | *Thunnus albacares* | XP_044212260.1 |
| Gene | species name | GenBank accession No |
| MTP18 | *Macrobrachium nipponense* | OR_209329.1 |
|  | *Penaeus chinensis* | XP_047476688.1 |
|  | *Penaeus vannamei* | XP_027217375.1 |
|  | *Penaeus japonicus* | XP_042857217.1 |
|  | *Portunus trituberculatus* | XP_045123380.1 |
|  | *Acipenser ruthenus* | XP_033902190.2 |
|  | *Salvelinus namaycush* | XP_038826422.1 |
|  | *Salmo trutta* | XP_029565293.1 |
|  | *Oncorhynchus keta* | XP_035631861.1 |
|  | *Dreissena polymorpha* | XP_052266022.1 |
|  | *Mizuhopecten yessoensis* | XP_021374394.1 |
|  | *Crassostrea angulata* | XP_052714411.1 |
|  | *Mytilus californianus* | XP_052073183.1 |
|  | *Rhagoletis pomonella* | XP_036343948.1 |
|  | *Lasius niger* | KMQ94477.1 |
|  | *Aedes albopictus* | XP_029707795.1 |
|  | *Anopheles albimanu* | XP_035782626.1 |

**Table S3.** Primers used in this study

| Primer | Primer sequence (5′-3′) |  |
| --- | --- | --- |
| **cDNA cloning** |  |  |
| DRP1-3′Race-out | AGGACAATCTCCAGAGCGAACTGG |  |
| DRP1-3′Race-in | TCGGAATCTCCAGAAATTGCCCTC |  |
| DRP1-5′Race-out | GCATCCATCAAATCCAGC |  |
| DRP1-5′Race-in | GCCAGAGTTCTCCGTCCA |  |
| MTP18-3′Race-out | CACCATCAATCGAGTGTGTGCAGCAT |  |
| MTP18-3′Race-in | ACCGCTCACAACTCGTAAATGGATAACC |  |
| MTP18-5′Race-out | ATGTCTGAAGTAAAGGAAGTGGAC |  |
| MTP18-5′Race-in | TTATTCCTCTTCCCTTGGAGCCAAG |  |
| **Promoter Plasmid construction** |  |  |
| MTP18-F1 (genome walking) | ACGGTGTTGAAGACATCCTG |  |
| MTP18-R1 (genome walking) | CTGTGGCAACTGGATGGC |  |
| MTP18-F2 (genome walking) | CTTTGGAAGATTGAGTGG |  |
| MTP18-R2 (genome walking) | GGCCTATTTATAACTACTTAATTTC |  |
| P1-F (promoter activity) -360~155 | AAAAACCTATCTAACTTTCCTTGCCG |  |
| P1-R (promoter activity) -360~155 | AAGCCCAGGCCCGAATTCGACGGAAAG |  |
| P2-F (promoter activity) -900~155 | TCTCTCTCTCTCTCTCTCTCTCTCCAC |  |
| P2-R (promoter activity) -900~155 | AAGCCCAGGCCCGAATTCGACGGAAAG |  |
| P3-F (promoter activity) -1350~155 | GAGAGATGTGTATTTCTGGTGATAGAAG |  |
| P3-R (promoter activity) -1350~155 | AAGCCCAGGCCCGAATTCGACGGAAAG |  |
| P4-F (promoter activity) -1752~155 | GCATAGATCTACACCAAGCCTCTTTTACCC |  |
| P4-R (promoter activity) -1752~155 | AAGCCCAGGCCCGAATTCGACGGAAAG |  |
| **RNA interference** |  |  |
| 1#siRNA- Drp1-F | GGAGAACUCUGGCAGUCAUTT |  |
| 1#siRNA- Drp1-R | AUGACUGCCAGAGUUCUCCTT |  |
| 2#siRNA- Drp1-F | GAGACAUGGUGUUGUCAUA |  |
| 2#siRNA- Drp1-R | UAUGACAACACCAUGUCUC |  |
| 1#dsRNA- Drp1-F | GAGCTCCGGTAAAAGCAGTG |  |
| 1#dsRNA- Drp1-R | GCATCCATCAAATCCAGCTT |  |
| 2#dsRNA- Drp1-F | GTCATCAACAAGCTGCAGGA |  |
| 2#dsRNA- Drp1-R | TTTGTAAGGCCAGGCAAATC |  |
| 1#dsRNA-MTP18-F | TGCATTCCTTTCATTGTCCA |  |
| 1#dsRNA-MTP18-R | TTTATAATGGGGCCTTGTGG |  |
| 2#dsRNA-MTP18-F | ATATGCAAATGAAGTCGGGG |  |
| 2#dsRNA-MTP18-R | AAGGAATGCACCCTAAGCCT |  |
| **Quantitative real-time PCR** |  |  |
| DRP1-F (real-time primer) | GTATCCATCCACTTGCTGGTCTCAC |  |
| DRP1-R (real-time primer) | GGTTCCTCAAGGCGTCTAATCTGTC |  |
| MTP18-F (real-time primer) | AGTGTGTGCAGCATCTTTGG |  |
| MTP18-R (real-time primer) | AGGAATGCACCCTAAGCCTATG |  |
| β-Actin-F (real-time primer) | TATGCACTTCCTCATGCCAT |  |
| β-Actin-R (real-time primer) | AGGAGGCGGCAGTGGTCAT |  |

**Supplementary Figures and legends**

**Figure S1**

**A Hypoxia B Hypoxia+Fer-1**


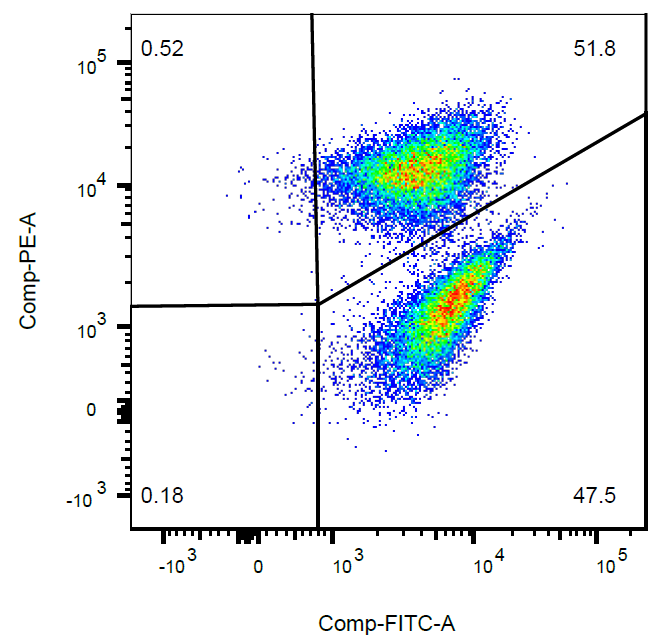

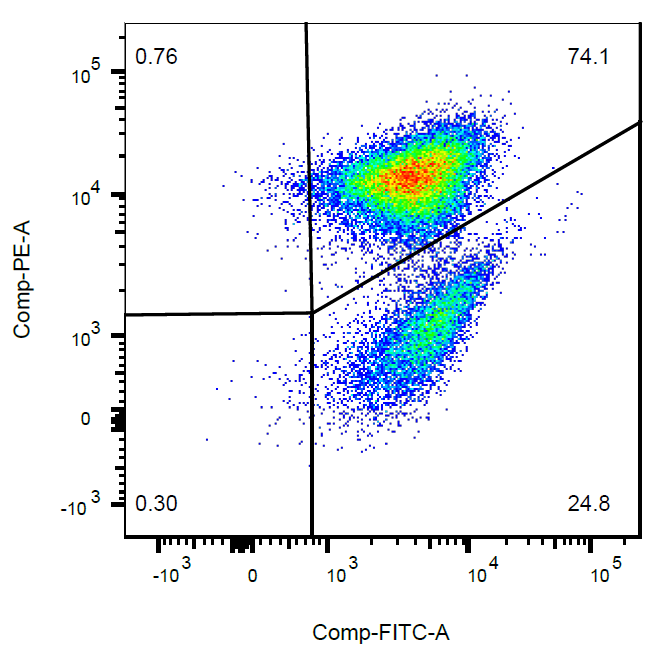


**C D**


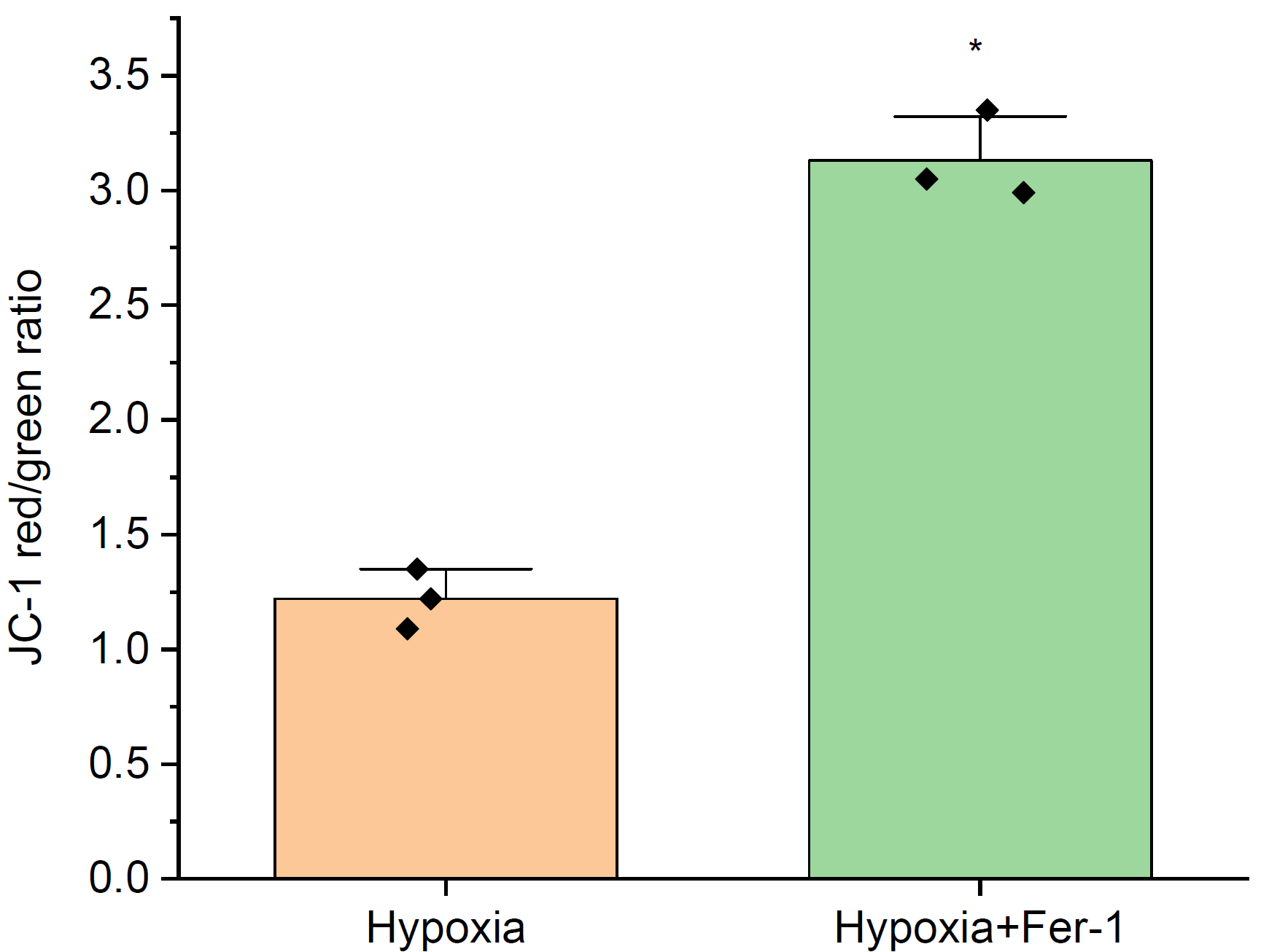

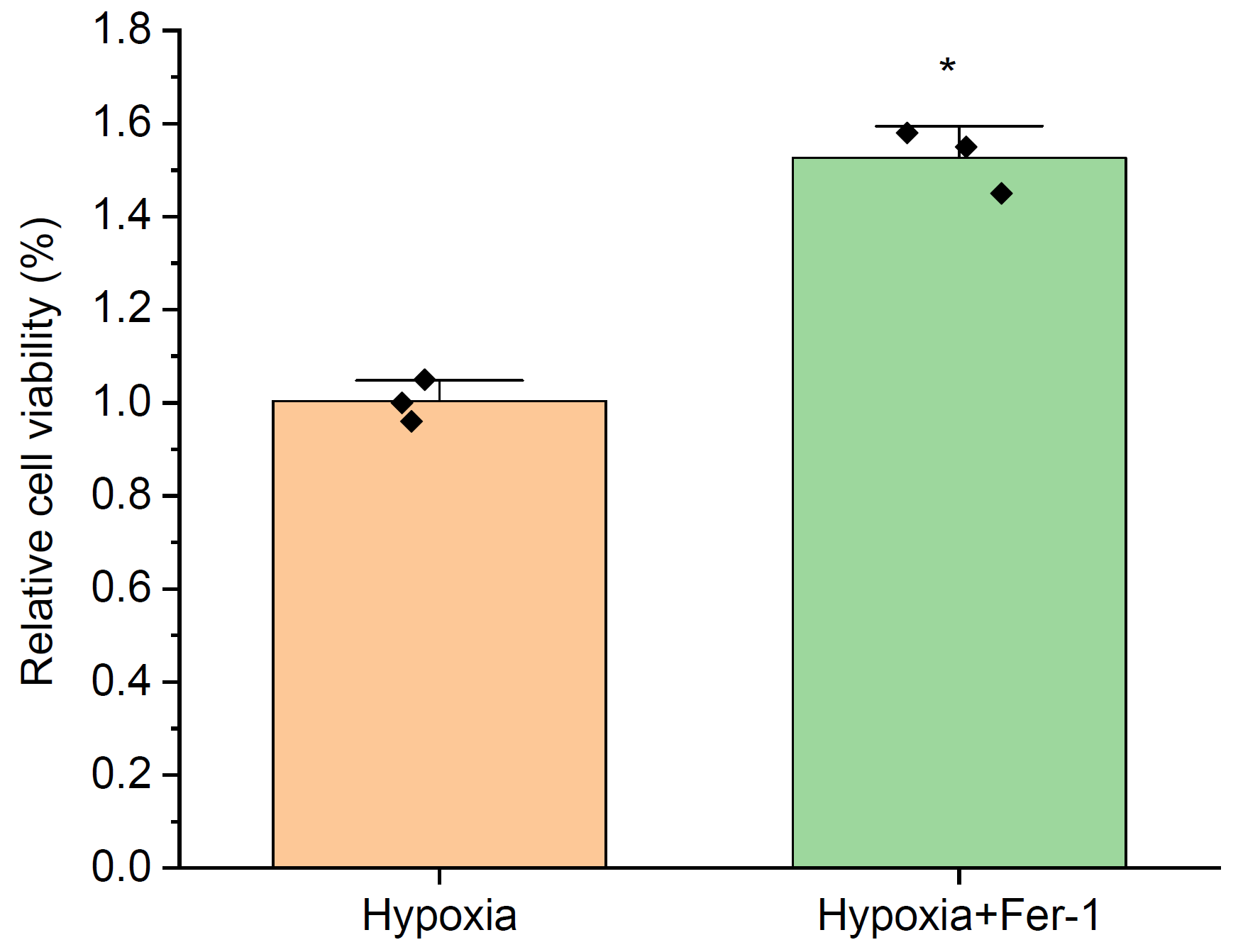


**Figure S1.** Ferrostatin-1 (Fer-1) as a ferroptosis inhibitors rescue hypoxia induced hemocyte ferroptosis. *A*-*C*, the effect of hypoxia along with Fer-1 on mitochondrial membrane potential. *D*, the effect of hypoxia along with Fer-1 on cell viability.

**Figure S2**


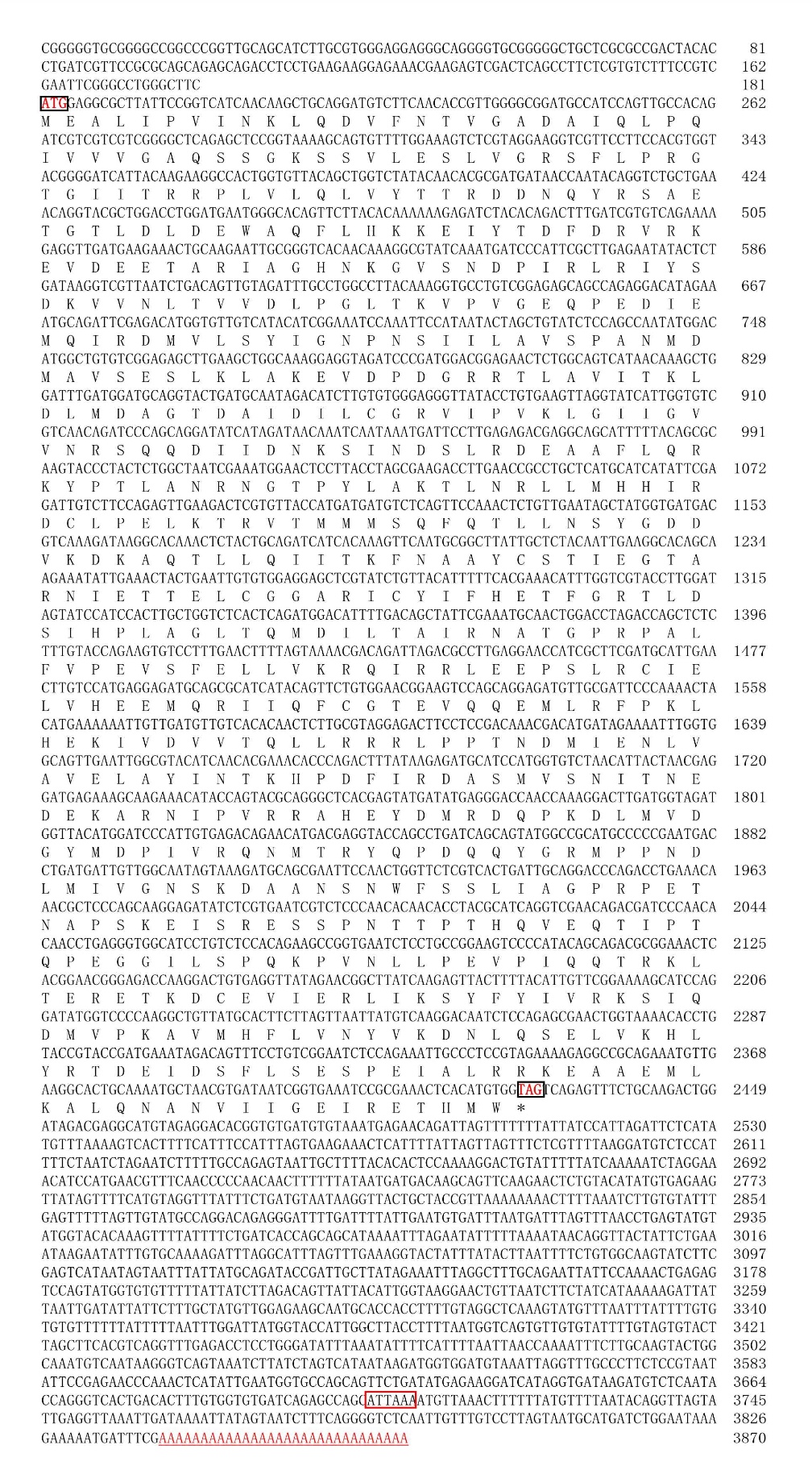


**Figure S2.** The deduced amino acid dynamin-related protein 1 (DRP1) sequence in *Macrobrachium nipponense* is displayed above the nucleotide sequence. The start codon (ATG) and stop codon (TAG) are marked with black boxes, respectively. The 3'-terminal poly(A) tailed signal (ATTAAA) is indicated by a red box, and the poly(A) tail is indicated by a red font and horizontal line.

**Figure S3**


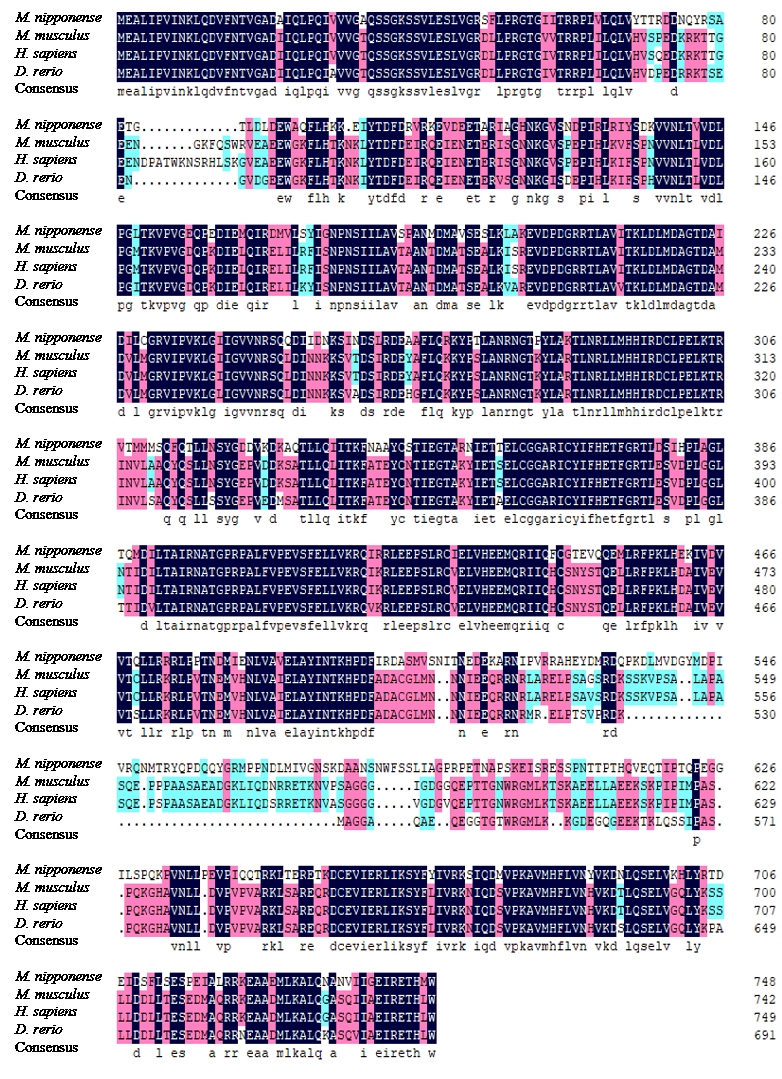


**Figure S3**. Multisequence alignment analysis of the Drp1 from *Macrobrachium nipponense* with homolog’s in other animals. Amino acid residues with 100% identity are shown in blue. Prediction Conserved domain GTPases are indicated by red boxes, stalk by black boxes, and GED by blue boxes.

**Figure S4**

**
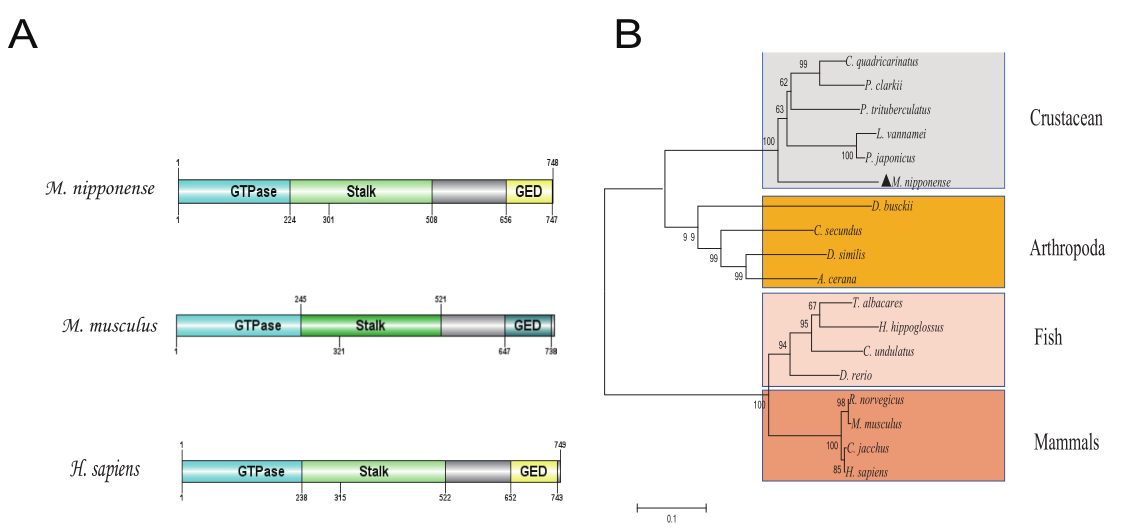
**

**Figure S4.** Evolutionary conservation of Drp1 in prawn. *A*, The domain organization of Drp1 in the oriental river prawn (*Macrobrachium nipponense*), mouse (*Mus musculus*), and human (*Homo sapiens*). *B*, A phylogenetic tree was constructed using the amino acid sequences of Drp1 from the indicated species. The phylogenetic tree was constructed using the neighbor-joining algorithm with the MEGA4 program, based on a multiple sequence alignment generated by ClustalW. Bootstrap values of 1000 replicates (percentages) are indicated on the branches. The accession numbers of the selected sequences are listed in Table S2.

**Figure S5**

**A B**

**C**


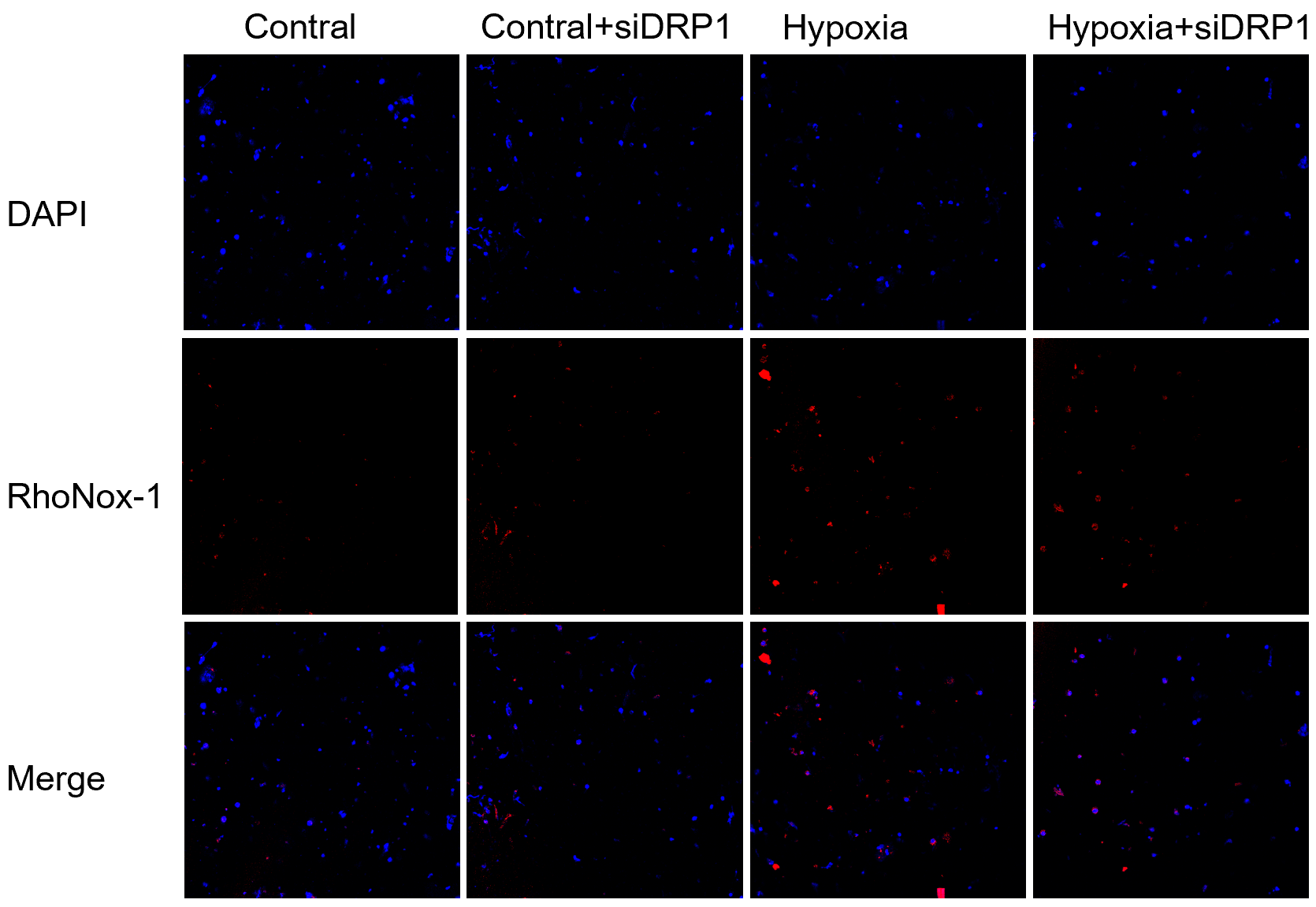


**Figure S5.** *Drp1* knockdown reduces ferroptotic stress emerges in hypoxic hemocytes *in vitro*. *A*, lipid peroxidation was assessed in hemocytes by flow cytometry using the fluorescent probes C11-BODIPY. *B*, MDA levels were measured to assess lipid peroxidation in hemocytes. *C*, Fe^2+^ levels were measured in hemocytes by flow cytometry using FeRhoNox-1 probe.

**Figure S6**


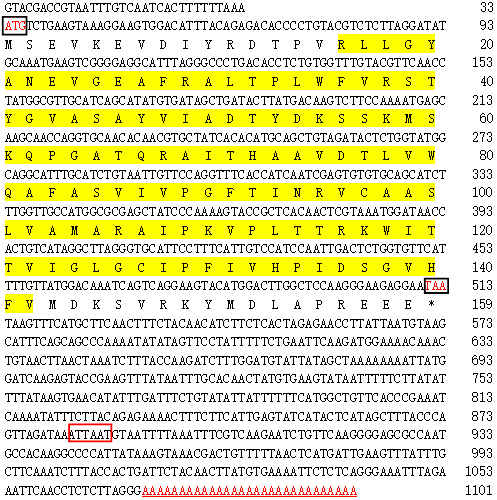


**Figure S6.** The deduced amino acid mitochondrial fission process protein 1-like (MTP18) sequence in *Macrobrachium nipponense* is displayed above the nucleotide sequence. The start codon (ATG) and stop codon (TAA) are marked with black boxes, respectively. The 3'-terminal poly(A) tailed signal (ATTAAA) is indicated by a red box, and the poly(A) tail is indicated by a red font and horizontal line. The structural domain MTP18 is indicated by a yellow underline.

**Figure S7**


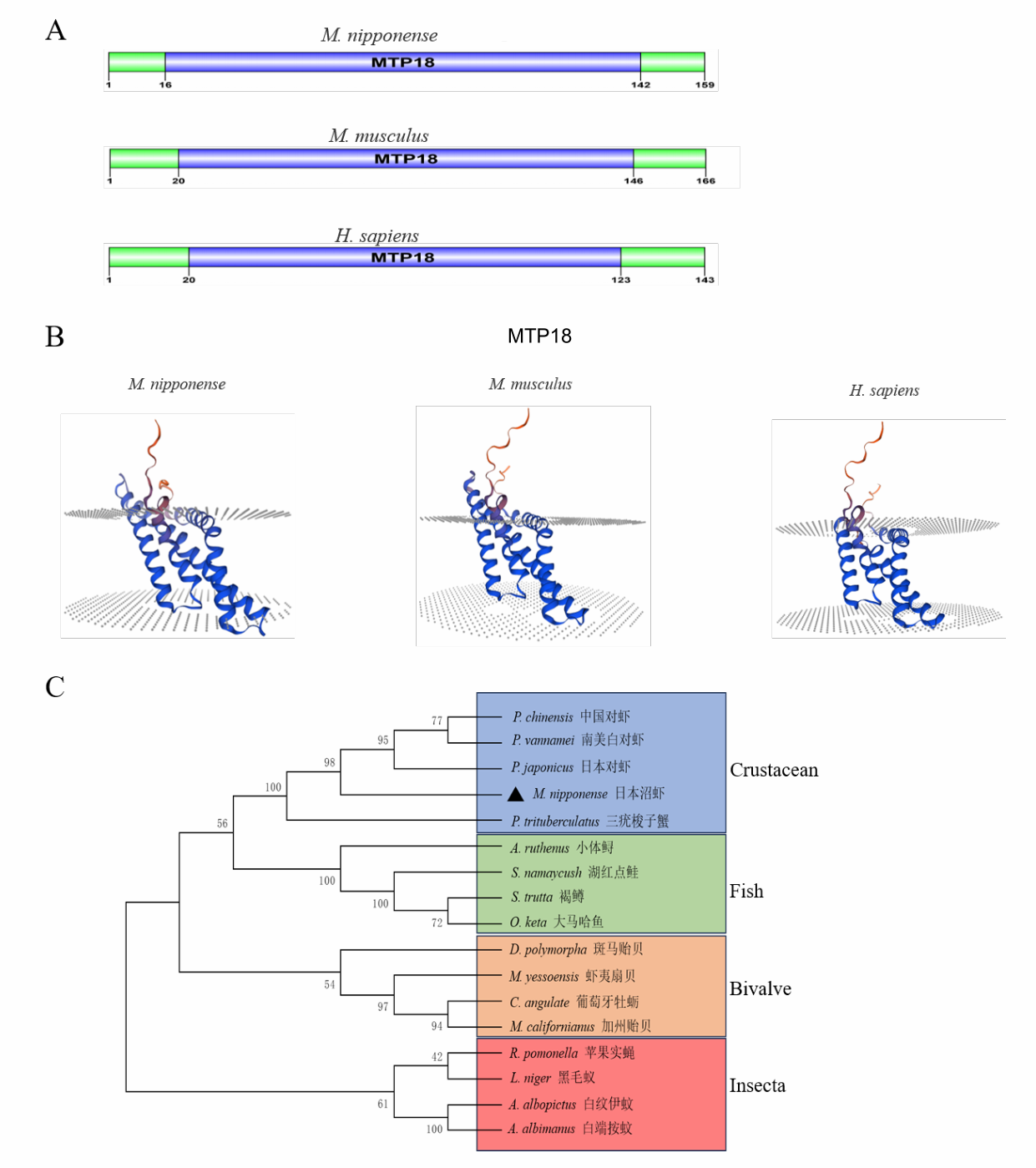


**Figure S7.** Evolutionary conservation of MTP18 in prawn. *A*, the domain organization of MTP18 in the oriental river prawn (*Macrobrachium nipponense*), mouse (*Mus musculus*), and human (*Homo sapiens*). Twelve conserved transmembrane domains are shown. *B*, prediction of the tertiary structures of prawn, mouse, and human MTP18 using SWISS-MODEL. *C*, a phylogenetic tree was constructed using the amino acid sequences of MTP18 from the indicated species. The phylogenetic tree was constructed using the neighbor-joining algorithm with the MEGA4 program, based on a multiple sequence alignment generated by ClustalW. Bootstrap values of 1000 replicates (percentages) are indicated on the branches. The accession numbers of the selected sequences are listed in Table S2.

**Figure S8**


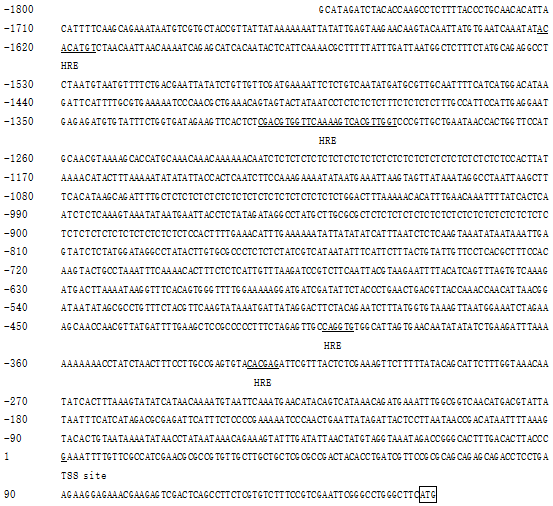


**Figure S8.** The 5’-flanking sequence of the MTP18 gene of the oriental river prawn. Putative transcription factor binding sites are underlined and labeled. Putative elements including HIF-1a binding sites (hypoxia response elements, HRE). The stop codon (TGA) was boxed.
